# Long Non-coding RNAs in Traumatic Brain Injury Accelerated Fracture Healing

**DOI:** 10.3389/fsurg.2021.663377

**Published:** 2021-06-04

**Authors:** Guoning Guo, Yajun Gou, Xingyu Jiang, Shuhong Wang, Ruilie Wang, Changqiang Liang, Guang Yang, Tinggang Wang, Anyong Yu, Guoyan Zhu

**Affiliations:** ^1^Department of Emergency, Affiliated Hospital of Zunyi Medical University, Guizhou, China; ^2^Department of Orthopedic, Shapingba District Hospital, Chongqing, China; ^3^Department of Health Management, Xinqiao Hospital, Third Military Medical University (Army Medical University), Chongqing, China

**Keywords:** traumatic brain injury, fracture, long non-coding RNAs, immune cells, endothelia cells

## Abstract

It is commonly observed that patients with bone fracture concomitant with traumatic brain injury (TBI) had significantly increased fracture healing, but the underlying mechanisms were not fully revealed. Long non-coding RNAs (lncRNAs) are known to play complicated roles in bone homeostasis, but their role in TBI accelerated fracture was rarely reported. The present study was designed to determine the role of lncRNAs in TBI accelerated fracture via transcriptome sequencing and further bioinformatics analyses. Blood samples from three fracture-only patients, three fracture concomitant with TBI patients, and three healthy controls were harvested and were subsequently subjected to transcriptome lncRNA sequencing. Differentially expressed genes were identified, and pathway enrichment was performed by Gene Ontology (GO) and Kyoto Encyclopedia of Genes and Genomes (KEGG) analysis. High-dimensional data visualization by self-organizing map (SOM) machine learning was applied to further interpret the data. An xCell method was then used to predict cellular behavior in all samples based on gene expression profiles, and an lncRNA–cell interaction network was generated. A total of 874 differentially expressed genes were identified, of which about 26% were lncRNAs. Those identified lncRNAs were mainly enriched on TBI-related and damage repair-related pathways. SOM analyses revealed that those differentially expressed lncRNAs could be divided into three major module implications and were mainly enriched on transcriptional regulation and immune-related signal pathways, which promote us to further explore cellular behaviors based on differentially expressed lncRNAs. We have predicted that basophils, CD8+ T effector memory cells, B cells, and naïve B cells were significantly downregulated, while microvascular endothelial cells were predicted to be significantly upregulated in the Fr/TBI group, was the lowest and highest, respectively. ENSG00000278905, ENSG00000240980, ENSG00000255670, and ENSG00000196634 were the most differentially expressed lncRNAs related to all changes of cellular behavior. The present study has revealed for the first time that several critical lncRNAs may participate in TBI accelerated fracture potentially via regulating cellular behaviors of basophils, cytotoxic T cells, B cells, and endothelial cells.

## Introduction

Fractures, the most common injuries of the musculoskeletal system of humans, are a major cause of global mortality and disability (1). Epidemiological investigation revealed that roughly 4.39 million Chinese have a traumatic fracture in 2014 (2). Fracture healing is a complex process that requires multiple cell involvement and signal transduction. Generally, as a mineralized tissue, bone was intended to resist tension, compression, and torsional stresses (1, 3). A fracture occurs when the deforming energy exceeds the maximum threshold of bone structure (4, 5). The initial response of bone injury was vascular disruption-induced hematoma and clot formation at the fracture site. This danger signal subsequently induces the infiltration of immune cells and the release of inflammatory cytokine, chemokines, and growth factors. This bone injury further activates stem/progenitor cells involved in bone regeneration at the periosteum and in the bone marrow. The stem/progenitor cells were then differentiated into chondrocyte lineage or osteoblast lineage to promote the formation of cartilage for repairing the injured bones. Cartilage callus tissue further undergoes calcification, and blood vessels invade so that bone could be formatted and remodeled secondarily (1, 5, 6). Although massive progress has been made for fracture treatment, ~10% of fractures do not heal as expected, termed as non-union. Other compromised fracture healing like delayed union and inappropriate tissue regeneration was also commonly seen in the clinic (5, 6). Therefore, the development of novel molecules for predicting or treating fracture that could be utilized in the clinic is still of great importance.

In clinical practice, we and other orthopedists found that accelerated fracture healing could be observed in patients with traumatic brain injury (TBI) (7–9). As previously reported, significantly accelerated callus formation was found in cases concomitant with TBI when compared with those in patients with fracture alone (7–9). The possible working mechanism of this clinical phenomenon was also little studied (8, 9). Currently, TBI accelerated fracture healing was observed to be highly associated with changes in mesenchymal stem cells, cytokines and growth factors, hormones, and other substances like calcium and phosphate (9). However, most of the abovementioned mechanisms were preliminary ones that originated from preclinical studies. There is still little known by which cytokines, growth factors, and hormones that mediated the signal pathway finally promote fracture healing.

Long non-coding RNAs (lncRNAs), a family of transcripts with strand length exceeding 200 bp that do not encode protein, were demonstrated to play critical roles in regulating gene transcription, epigenetic regulation, cell proliferation, and cell differentiation (10–12). According to GENCODE annotation for humans, over 16,000 lncRNAs were identified, and those lncRNAs were reported to participate in numerous diseases and were not considered as novel targets/markers for disease diagnosis, treatment, or prognosis prediction. As previously reported, lncRNAs were vital in maintaining bone homeostasis since they could regulate the expression of key enzymes and proteins in the process of bone formation and resorption. For example, H19, an upregulated lncRNA during osteogenesis in bone mesenchymal stem cells (BMSCs), together with forkhead box C2, was proved to have the ability to promote osteogenic differentiation of BMSCs (13). Another lncRNA, differentiation antagonizing non-protein coding RNA (DANCR), was also demonstrated to inhibit osteogenic differentiation of human BMSCs (14). In animal models of fracture, LOC103691336 was reported to modulate osteogenic differentiation in a rat femoral fracture model (15); downregulated lncRNA-ANCR was found to promote tibial fracture healing in rabbits by regulating the expression of runt-related transcription factor 2 (RUNX2) (16); in mouse tibia fracture models, silencing or inhibiting of maternally expressed gene 3 (MEG3) (17) or TSIX (18) was proved to accelerate fracture healing. However, the role of lncRNAs in TBI accelerated fracture healing has never been reported before. The present study was designed to identify differentially expressed lncRNA via high-throughput sequencing and to further determine their roles in TBI accelerated fracture healing by bioinformatics analysis. The present study might form the experimental basis of potential targets of lncRNA for fracture treatment.

## Patients and Methods

### Demographic Data

The research protocol was approved by the ethical committee of Affiliated Hospital of Zunyi Medical University and was performed in compliance with the Declaration of Helsinki. Approximately 30 TBI patients, 100 fracture patients, and 50 TBI concomitant with fracture patients are admitted into our hospital monthly. Due to funding limitations, the sample size for this study was limited to three TBI concomitant with fracture patients. These were randomly selected in accordance with the method of random number table based on the inclusion and exclusion criteria. Likewise, three patients matched for age, gender, and fracture sites without concomitant TBI were also selected in accordance with the method of random number table. To avoid potential selection bias, randomization of selection was performed by a member of the study team who is not on the staff of our hospital. Three fracture-only patients (two male and one female, average age was 45.33 ± 6.51 years, defined as the FrO group) and three fracture concomitant with TBI patients (two male and one female, average age was 46.67 ± 2.88 years, defined as the Fr/TBI group) admitted in the Department of Emergency, Affiliated Hospital of Zunyi Medical University from January 2019 to March 2019, were involved in the current research (as presented in Table 1). Three age- and sex-matched healthy controls (two male and one female, average age was 47.33 ± 6.11 years, defined as HC) were all medical staff from our department. Patients in the Fr/TBI group have significantly accelerated fracture healing time than those patients in the FrO group (Table 1). Participants involved in the current study have signed an informed consent form.

**Table 1:** Demographic data.

	**HC** ** (*N* = 3)**	**FrO** ** (*N* = 3)**	**Fr/TBI** ** (*N* = 3)**
Age	47.33 ± 6.11	45.33 ± 6.51	46.67 ± 2.88
Gender (male, *N*)	2	2	2
Fracture/TBI	NA	Patients #1: Right humerus fracture; Patients #2: Left femoral fracture; Patients #3: Right tibial fracture;	Patients #1: Right tibial fracture with right frontal lobe contusion and laceration; Patients #2: Left femoral fracture with left temporal lobe contusion and laceration; Patients #3: Right humerus fracture with bilateral frontal lobe brain contusion;
Fracture healing time (Weeks)	NA	11.57 ± 0.80	9.37 ± 0.96^*^

* *P <0.05*.

Fractures were diagnosed by X-ray photography and computed tomography (CT). All fractures were treated by surgery with internal fixation as performed by the same surgeon. TBI was diagnosed by CT, and the severity of TBI was evaluated by Glasgow Coma Scale (GCS). All TBI patients were treated with individualized protocol based on patients' conditions. Fracture healing time was recorded when bone union emerged as determined by X-ray photography.

### Inclusion and Exclusion Criteria

Patients of the FrO or Fr/TBI group were included or excluded by the following criteria. Inclusion criteria: (1) clear fracture determined by X-ray photography and CT; (2) normal functions in critical organs like the heart (normal cardiac function as determined by electrocardiography and echocardiography), liver (indicated by serum biochemical tests), and kidney (indicated by serum and urine biochemical tests); and (3) GCS score of 9 to 12 for TBI patients. Exclusion criteria: (1) with severe infection complications; (2) immunodeficiency; (3) complications with other acute or chronic diseases, like cardiovascular diseases and metabolic diseases; (4) severe TBI (GCS score of 8 or less); and 5) severe open fractures (type II and III Gustilo open fracture), or transverse fractures.

### Long Non-coding RNA High-Throughput Sequencing

Blood samples were harvested 7 days after admission. Total RNA from 4 ml of peripheral whole blood of each sample was extracted by 12 ml of TRIzol reagent according to manufacturer's protocols. Purity, concentration, and integrity of isolated RNA were further determined or verified to make sure the isolated total RNA was suitable for further analyses. Three micrograms of total RNA per sample was used to generate sequencing libraries after removal of ribosomal RNA. After cluster generation, the harvested libraries were sequenced on an Illumina HiSeq platform. Raw data of sequenced RNA in FASTAQ format were then subjected to quality control, and clean data were generated for further downstream analyses. Assembled transcripts were then annotated to reference genomics.

### Gene Ontology and Kyoto Encyclopedia of Genes and Genomes Analyses

Differentially expressed genes or lncRNA target genes were subjected to Gene Ontology (GO) enrichment analysis by using clusterProfiler (19) R package. GO terms with a corrected *P*-value < 0.05 were considered to be significantly enriched.

Kyoto Encyclopedia of Genes and Genomes (KEGG) is a database resource for understanding high-level functions and utilities of a biological system, at the cell, organism, and ecosystem levels, using molecular-level information, especially large-scale molecular datasets generated by genome sequencing and other high-throughput experimental technologies (http://www.genome.jp/kegg/). An R package of clusterProfiler was used to determine the enrichment of differentially expressed genes or lncRNA target genes in KEGG pathways.

### High-Dimensional Data Portraying by Self-Organizing Map Machine Learning

To better interpret harvested data, a machine learning-based self-organizing map (SOM) was used by utilizing R package of oposSOM (20, 21) (v2.8.0). The oposSOM was able to translate RNA-seq expression data into metadata of a reduced dimension. This package can provide various sample-centered and group-centered visualizations, sample similarity analyses, and functional enrichment analyses. In the current study, we adopted a sample-centered visualization of harvested lncRNAs, and map size was selected in accordance with recommendations from the developer. Pre-processed expression matrix was presented in a log scale and then subjected to SOM training from different gene expression matrices. Generally, a cluster of genes with similar expression profiles was taken as a metagene, which was selected by SOM training. The training process was terminated until all expression patterns in the data matrix metagenes were captured by metagenes. In the current study, a total of 10 expression modules were harvested and labeled from A to J based on K-means cluster. Neighbor-joining was used to obtain a dendrogram.

### Inference of Infiltrating Cells From Gene Expression Profiles

To determine the fraction of infiltrated cells, we used xCell (22), which was a computational method based on ssGSEA and provides immune scores for 64 cell subtypes, spanning multiple innate and adaptive immune cells. It employs a spillover compensation technique to reduce dependencies between closely related cell types.

### Long Non-coding RNA–Cell Interaction Network and Long Non-coding RNA–mRNA Co-expression Network Construction

Spearman's correlation analysis was applied to calculate the cor-relationship between the expression of lncRNAs and the changes of cellular behavior. Pearson's correlation analysis was used to calculate the cor-relationship between the expression of lncRNAs and mRNAs. |Correlation coefficient| > 0.5 and *P* < 0.05 was considered to be interdependent. Cytoscape (version 3.6.2) was used to present the network.

### Statistical Analysis

All computational and statistical analyses were performed using R (version 3.5.1). An unpaired Student's *t*-test or Wilcoxon's test was used to compare the differences between two groups. One-way ANOVA was used to analyze the differences among three or more groups. *P* < 0.05 was considered statistically significant.

## Results

### Expression Profile of Sequence Analysis

**Figure 1:**
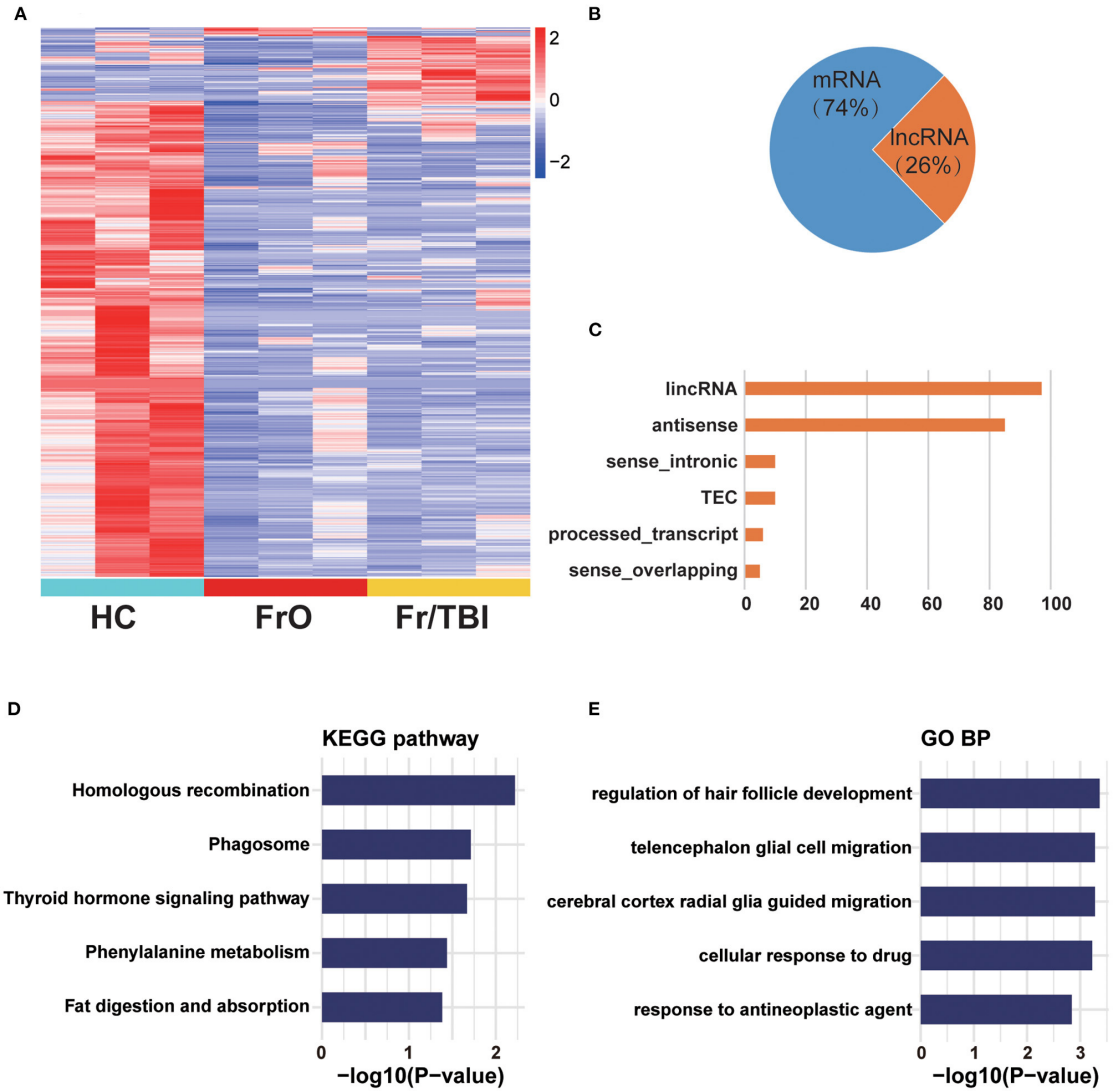
Expression profile of sequence analysis. **(A)** Heatmap showed all differentially expressed genes from healthy volunteers and patients. **(B)** Composition of identified differentially expressed genes from long non-coding RNA (lncRNA) sequencing. **(C)** Counts of each lncRNA subtype of identified lncRNAs. lincRNA, large intergenic non-coding RNA; TEC, to be experimentally confirmed lncRNA. **(D)** Kyoto Encyclopedia of Genes and Genomes (KEGG) enrichment analysis of differentially expressed genes. **(E)** Gene Ontology (GO) enrichment analysis of differentially expressed genes.

To determine whether expressions of lncRNAs were changed in TBI accelerated fracture, an lncRNA-based transcriptome sequencing was performed. As presented in Figure 1, we identified a total of 874 genes that were differentially expressed among the three groups (Supplementary Table 1). Of all identified genes, only about 26% were lncRNAs. Among all identified lncRNAs (Figure 1B, Supplementary Table 1), the majority were large intergenic non-coding RNAs (lincRNAs) and antisense lncRNAs (Figure 1C). These differentially expressed mRNAs and lncRNAs were mainly involved in TBI-related and damage repair-related pathways (like homologous recombination and phagosome pathway analyzed by KEGG; as well as regulation of hair follicle development and telencephalon glial cell migration biological process analyzed by GO, Figures 1D,E).

### Self-Organizing Map–Portrayal Expression Maps

**Figure 2:**
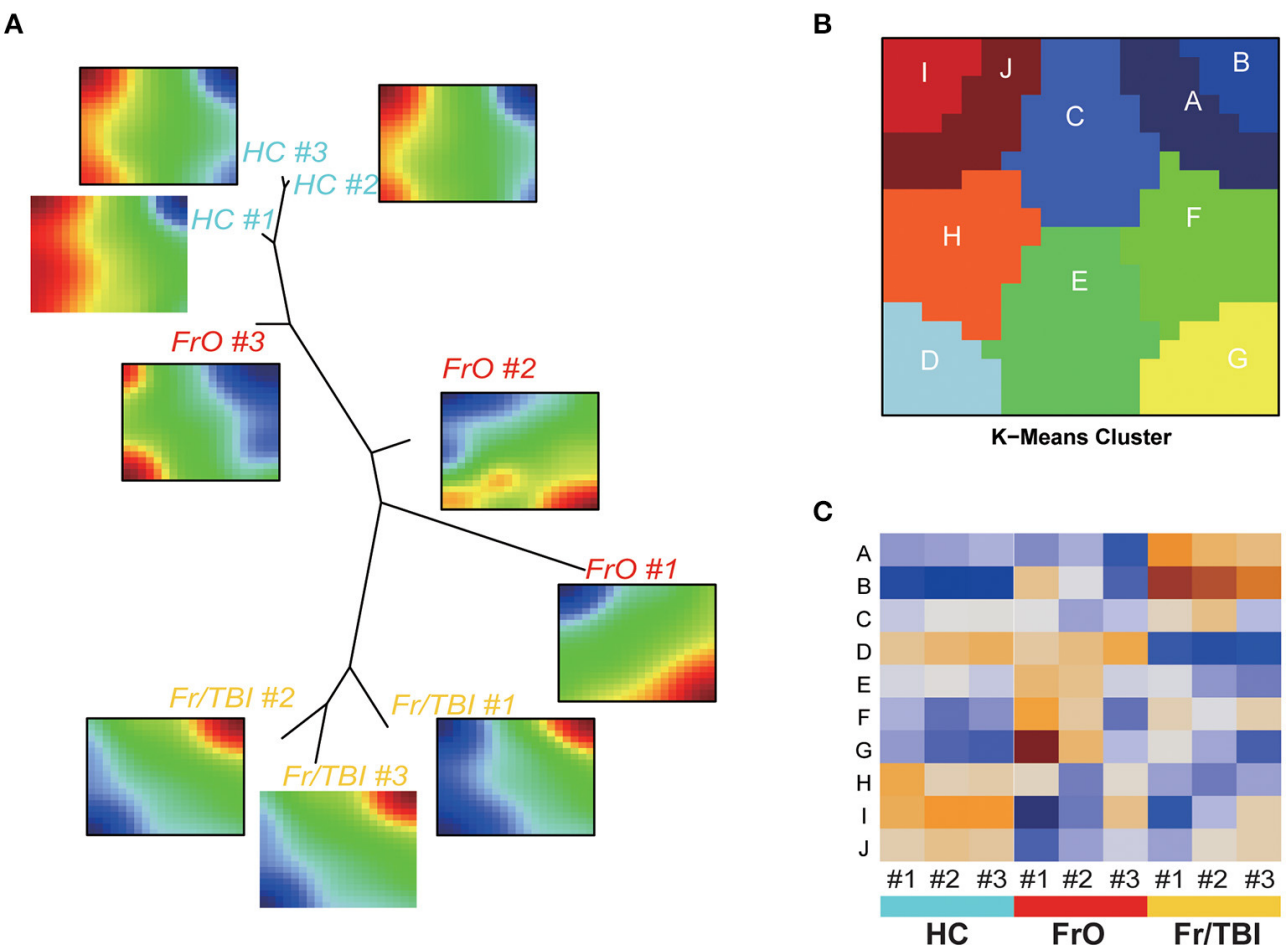
Self-organizing map (SOM)–portrayal expression maps. **(A)** Representative image of SOM analyses of the expression pattern of each sample. Dendrogram summarized the similarity of samples. **(B)** Representative image of gene expression modules. **(C)** Heatmap of expressions of modules in each sample.

As presented in the heatmap, a significantly changed expression pattern could be observed. To better interpret the sequencing results, we adopted a SOM machine learning-based data portraying method, which generated in a sample-centered manner. By using the neighbor-joining method based on the result of SOM, a dendrogram was obtained, which showed the state of development among the three groups, as expected (Figure 2A). To better identify the key different genes among the three groups, a total of 10 modules were generated based on similarities of expression profiles by K-means cluster (Figure 2B, Supplementary Table 1). Further analyses showed the expression pattern of each module (Figure 2C).

### Annotation of Harvested Expression Modules

**Figure 3:**
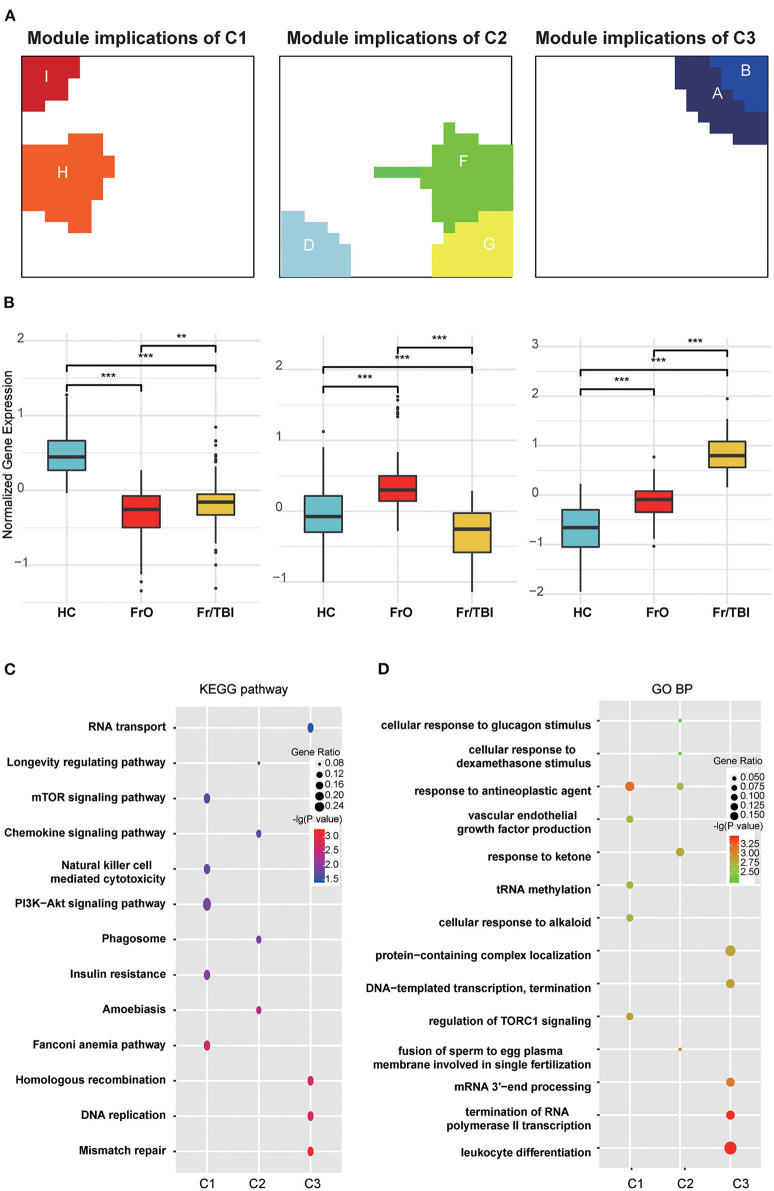
Annotation of harvested expression modules. **(A)** Representative images of module implications subdivision. **(B)** Boxplot showed the normalized gene expressions of subdivided module implications in each group. *P*-values were obtained via Wilcoxon's test. ** and *** indicate *P*-value < 0.01 and *P*-value < 0.001, respectively. **(C)** Kyoto Encyclopedia of Genes and Genomes (KEGG) enrichment analyses of genes from each module implication. **(D)** Gene Ontology (GO) biological process enrichment analyses of genes from each module implication. The vertical and horizontal axes represent the KEGG pathways and different modules, respectively. The size and the color intensity of a circle represent gene number and –log10 (*P*-value), respectively.

According to the expression pattern of each module, the harvested modules were further divided into three groups (modules H and I were defined as module implications of C1; modules D, F, and G were defined as module implications of C2; modules A and B were defined as module implications of C3; while no expression changes were observed in modules C and E). In all subdivided module implications, normalized gene expressions were significantly downregulated or upregulated among the investigated groups. In module implications of C1, normalized gene expressions in FrO group were the lowest, while in the HC group, they were the highest (Figures 3A,B). In module implications of C2, normalized gene expressions in FrO group were the highest, while in the Fr/TBI group, they were the lowest (Figures 3A,B). In module implications of C3, normalized gene expressions in the HC group were the lowest, while in Fr/TBI group, they were the highest (Figures 3A,B). Further pathway enrichment analyses by KEGG revealed that top enriched pathways were transcriptional regulation and immune-related signal pathways (like the Fanconi anemia pathway, amoebiasis, and mismatch repair) in all subdivided module implications (Figure 3C). GO analysis also revealed the same tendency; as proved by the top enriched biological process, there was leukocyte differentiation in all subdivided module implications (Figure 3D).

### Predicted Cellular Behavior

**Figure 4:**
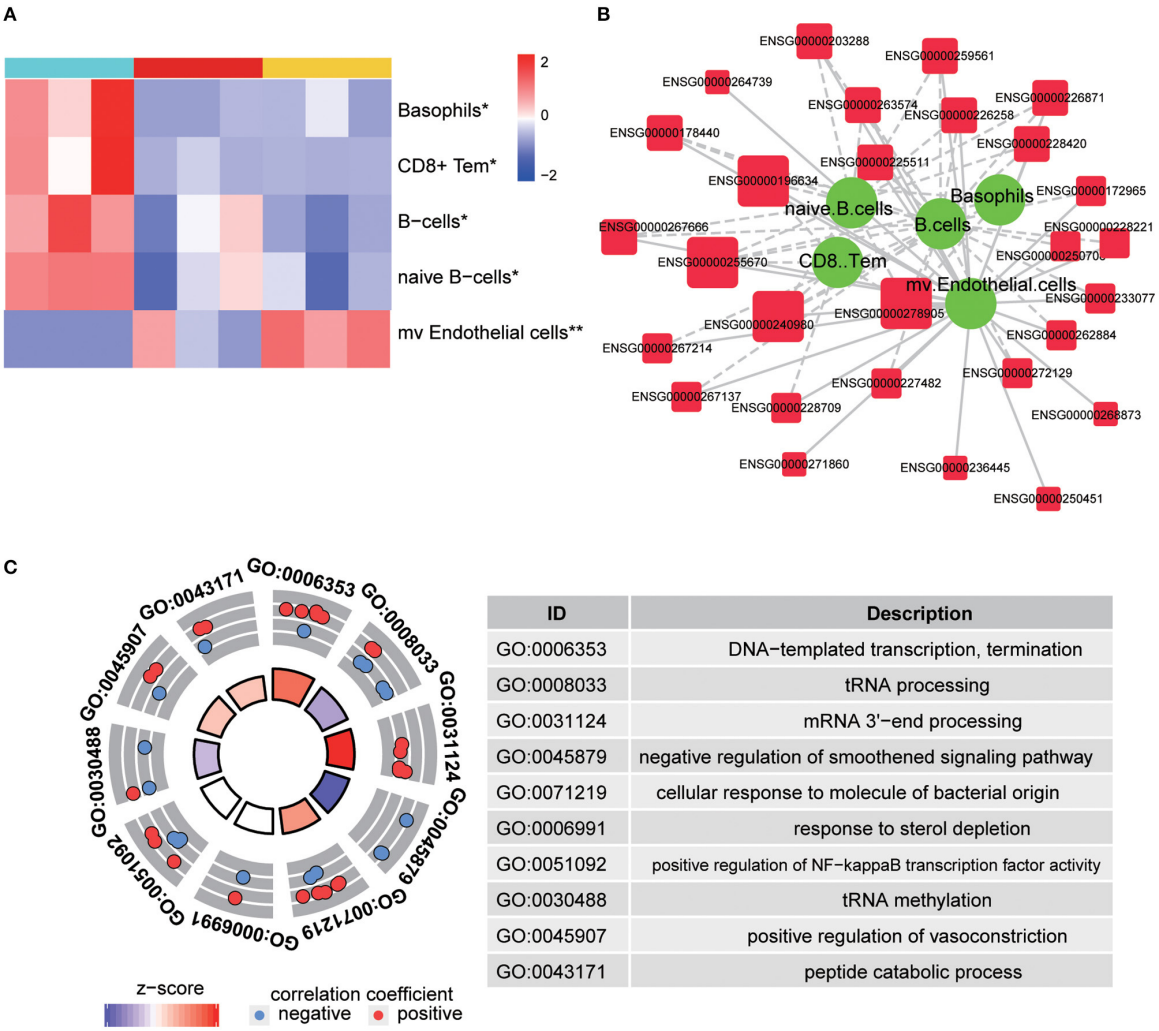
Predicted cellular behavior. **(A)** Representative heatmap is shown to predict significantly changed cellular behaviors in each group. *P*-values were obtained via one-way ANOVA; * and ** indicate *P*-value < 0.05 and *P*-value < 0.01, respectively. CD8+ Tem, CD8+ effector memory T-cells; mv endothelial cells, microvascular endothelial cells. **(B)** Representative image of interactions between identified long non-coding RNA (lncRNA) and changed cellular behavior. The larger size of lncRNA node indicates that more types of changed cellular behavior correlated with it. The thickness of a line represents the correlation coefficient. Solid and dotted lines showed the positive and negative relationships between the expression and changed cellular behavior, respectively. **(C)** Gene Ontology (GO) enrichment analysis of changed cellular behavior related to lncRNAs. The outer circle shows a scatter plot for each term of the correlation coefficient of the assigned target genes, and the *z*-score shows the number of positive genes minus the number of negative genes divided by the square root of the count.

The abovementioned enrichment analysis led us further to determine the cellular response in TBI accelerated fracture healing. We have predicted several changed cellular behaviors, as basophils, CD8^+^ T effector memory cells, B cells, and naïve B cells were predicted to be significantly downregulated, while microvascular endothelial cells were predicted to be significantly upregulated in the Fr/TBI group, which were the lowest and highest, respectively (Figure 4A and Supplementary Table 2). Correlation analyses were utilized to determine the relationship between differentially expressed lncRNA and known cellular behavior, and we created an lncRNA–cell interaction network based on lncRNA from C3 (Figure 4B). The result revealed that most expressions of lncRNAs from C3 were correlated with these changes of cellular behavior (Supplementary Table 3). In addition, ENSG00000278905, ENSG00000240980, ENSG00000255670, and ENSG00000196634 were the most differentially expressed lncRNAs that related to changes of cellular behavior. As indicated in Genotype-Tissue Expression Project (GTEx, https://gtexportal.org/home/), those four lncRNAs were expressed in various human tissues (Supplementary Figure 1). To predict the potential function and targets of these four lncRNAs, we constructed lncRNA–mRNA co-expression network, and then GO analysis (Figure 4C) of this network revealed that the top four cellular behavior-related lncRNAs were enriched mainly on transcriptional regulation and immune-related biological process, suggesting that those lncRNAs may participate in TBI accelerated fracture healing via regulating immune-related signaling potentially through transcriptional regulation.

## Discussion

The presented results have revealed for the first time that lncRNAs may be important in regulating TBI accelerated fracture healing. It was reported that lncRNAs could be involved in the maintenance of bone homeostasis via regulating chromatin remodeling, transcriptional activation/repression, post-transcriptional modification, or interaction with microRNAs (10–12). Currently, the relationship of lncRNAs and fracture healing was not well-acknowledged, and lncRNAs were reported to participate in fracture healing through interaction with microRNAs or by regulating protein expression without fully clarified mechanisms (13–18). In the present study, we have identified four major lncRNAs, namely, ENSG00000278905 (AC106818.1), ENSG00000240980 (AC093904.1), ENSG00000255670 (AC007619.1), and ENSG00000196634 (LUADT1), which may critically participate in TBI accelerated fracture healing. Except for LUADT1, the other three were newly identified ones that may be involved in fracture healing and have not been reported before. Also, little is known about the specific role of LUADT1 in this process since its role was mainly studied in tumorigenesis (23). Current reports reveal that the role of LUADT1 in bone homeostasis was performed to uncover its role in the behavior of primary chondrocytes (24). Those researchers have found that LUADT1 could participate in chondrocyte apoptosis by regulating miR-34a/SIRT1 signal pathway (24). Therefore, the specific role of those four newly identified lncRNAs in fracture healing remains to be explored.

Although fracture healing is a complex process, the course of fracture could be divided into three partially overlapping phases, namely, inflammation, repair, and remodeling (3). Fracture-induced blood vessel rupture would result in hematoma (characterized by hypoxia and low pH), which further initiates immune cascades and promotes the immune cell infiltration to injury sites. According to published literature, the first accumulated immune cells are polymorphonuclear neutrophils (PMNs) (25) that attracted debris and signals from cell death. The short-lived PMNs were reported to secrete massive cytokines and chemokines [C-C motif chemokine 2 (CCL2), IL6, TNF-α, etc.] to subsequently promote the infiltration of other immune cells like macrophages and other lymphocytes (26, 27). In a rodent model of fracture, systematic PMN activation could impair fracture healing (28). Also, a report has found that enhanced fracture healing could result in neutropenia in animal models of fracture (29). In the present study, we have found that basophils were predicted to be significantly decreased in TBI accelerated fracture patients, which is consistent with previous findings. The macrophages infiltrated from local environment (osteomacs) or from circulation (inflammatory macrophage) promote intramembranous bone formation and regulate the process of endochondral ossification (30). In the current study, we have not found any predicted quality/quantity changes associated with macrophages, potentially suggesting that macrophages participate in TBI accelerated fracture at the current time setting.

Behaviors of T cells and B cells also alter the process of fracture healing. In a mouse model of fracture, recombination activating gene 1 knockout mouse (Rag-1^−/−^, and T- and B-cell deficiency) showed delayed cartilage maturation and less mineralized tissue (31). However, another group has found that deficiency in the adaptive immune system could result in accelerated fracture healing (32). In a sheep fracture model, cytotoxic T cells (CD8^+^ T cells) were accumulated in a considerably increased abundance in delayed fracture healing group accompanied with enhanced inflammation (33), while in a rat model of fracture, depletion of cytotoxic T cells by neutralizing antibody did not affect bone formation by reducing the implant pull-out force and stiffness (34). It was reported that B cells can secrete osteoprotegerin to inhibit osteoclastogenesis to maintain bone homeostasis. The frequency of naïve B cells was decreased while plasmablasts were increased immediately after fracture, which suggested an inflammatory status of the body after fracture (35). At the remodeling phase of fracture healing, B cells with regulatory function critically affect tibia fracture healing as loss of B-cell regulatory function was observed to be associated with delayed healing in patients (36). In our current study, we have found that cytotoxic T cells, B cells, and naïve B cells were predicted to significantly decrease in patients of TBI accelerated fracture, which was not quite consistent with the previous findings. This paradoxical phenomenon could be explained by the following researches: on the one hand, cyclooxygenase-2 (COX-2) inhibitors (non-steroidal anti-inflammatory drugs) could significantly impair fracture healing (37); on the other hand, patients with diabetes mellitus (systematic inflammation) also have impaired fracture healing (38). It seems like a certain degree of inflammation is important in promoting fracture healing, while to what extent the inflammation is beneficial remains to be answered.

Another critical step of fracture healing is revascularization, which provides a structural basis for nutrient supply and osteoblast lineage fate specification (5). Endothelial cells were the major component of the neovascular. The interaction between those endothelial cells and osteoprogenitors would provide signals for osteoblast generation (39). Especially, type H endothelial cells not only were able to promote angiogenesis (40) but also could enhance osteogenesis through Notch signaling (41). In our current study, we have found that microvascular endothelial cells were predicted to be significantly enriched in patients of TBI accelerated fracture when compared with other groups, which was parallel with the positive role of endothelial cells in fracture healing.

This study has several limitations. Firstly, due to sample availability and funding limitation, the present study only includes three patients in each group, and sequencing was not performed on patients with TBI alone. Secondly, the diagnostic or prognostic value of identified lncRNAs was not validated in the current study. Thirdly, the source of identified lncRNAs was not determined. Fourthly, identified lncRNAs in the current study were not fully investigated to determine whether they were consequences or reasons of TBI accelerated fracture. Finally, whether severity of TBI affects fracture healing in a different manner was also not included in the current study.

## Conclusions

The present study has identified several lncRNAs that may participate in TBI accelerated fracture via regulating behaviors of basophils, cytotoxic T cells, B cells, and endothelial cells. However, massive experimental validations were strongly encouraged to promote the reliability of the current study.

## Data Availability Statement

The data presented in the study are deposited in the Gene Expression Omnibus repository (https://www.ncbi.nlm.nih.gov/geo/), accession number GSE171718.

## Ethics Statement

The studies involving human participants were reviewed and approved by Ethical Committee of Affiliated Hospital of Zunyi Medical University. The patients/participants provided their written informed consent to participate in this study. Written informed consent was obtained from the individual(s) for the publication of any potentially identifiable images or data included in this article.

## Author Contributions

GG, TW, GZ, and AY designed this research project and drafted and revised the manuscript. GG, YG, XJ, SW, GZ, and RW carried out the experiments, analyzed the data and drafted the manuscript. CL, GZ, and GY checked the analysis and revised the manuscript. All authors reviewed the manuscript and approved the manuscript that was submitted for publication.

## Conflict of Interest

The authors declare that the research was conducted in the absence of any commercial or financial relationships that could be construed as a potential conflict of interest.
