# Supplementary material for: Long Non-coding RNAs in Traumatic Brain Injury Accelerated Fracture Healing
Source: Front Surg. 2021 Jun 4;8:663377. doi: 10.3389/fsurg.2021.663377 (PMC8211774; doi:10.3389/fsurg.2021.663377)
Supplement: Supplementary file 1 [file Data_Sheet_1.docx]

Supplementary Material

# Supplementary Figures and Tables

## Supplementary Figures. Results of Genotype-Tissue Expression (GTEx) project show that three of the four identified lncRNAs is widely expressed in tissues of healthy individuals while ENSG00000240980 is expressed only in normal testis tissues.


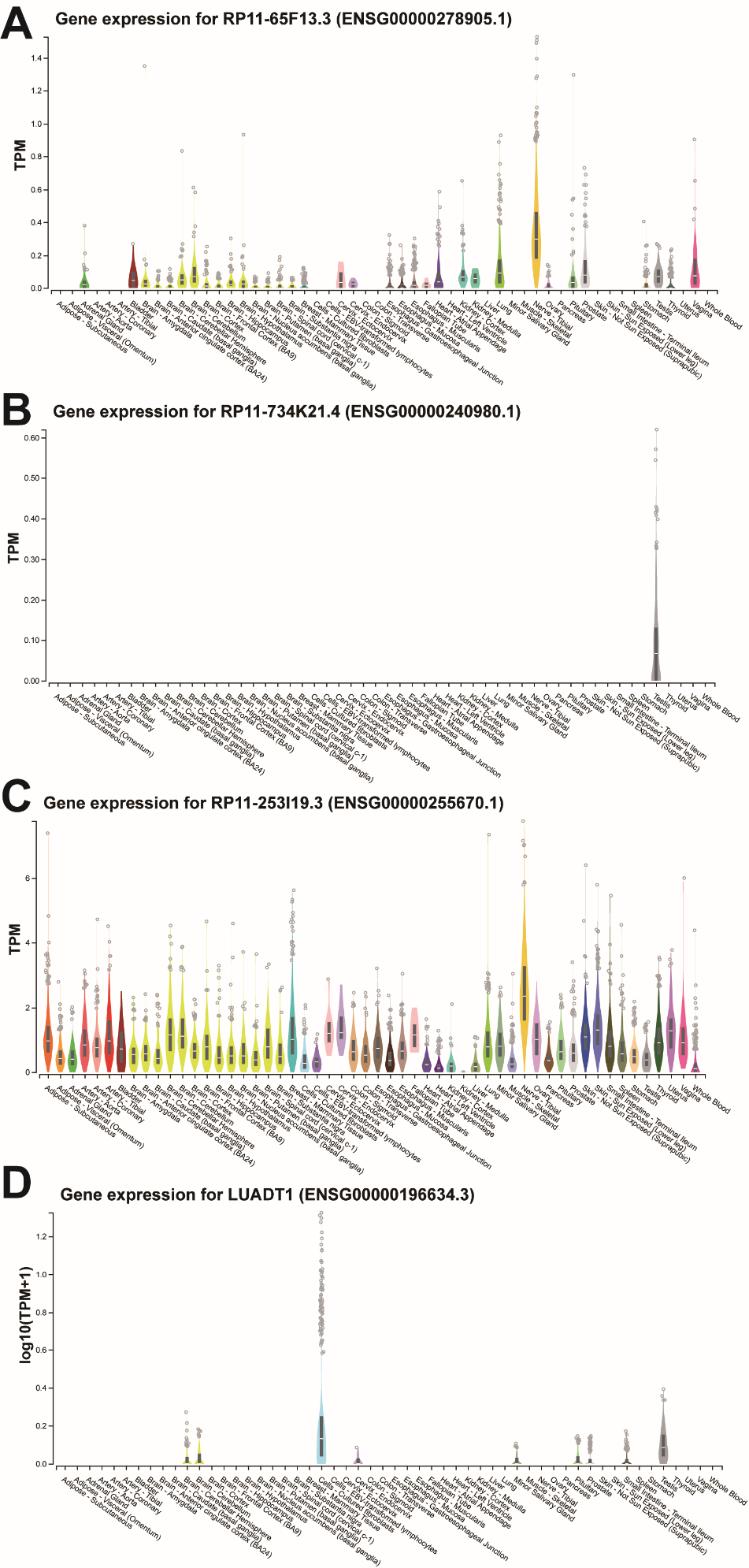


**Supplementary Figure legends.** Normal expression patterns of ENSG00000278905, ENSG00000240980, ENSG00000255670, and ENSG00000196634 in human tissues and cells as determined by GTEx project.

## Supplementary Tables

**Supplementary Table 1.** Expression modules generated by on K-means cluster.

| ID | Modules | Type |
| --- | --- | --- |
| ENSG00000077514 | A | mRNA |
| ENSG00000103227 | A | mRNA |
| ENSG00000106565 | A | mRNA |
| ENSG00000108417 | A | mRNA |
| ENSG00000131015 | A | mRNA |
| ENSG00000131059 | A | mRNA |
| ENSG00000143324 | A | mRNA |
| ENSG00000150457 | A | mRNA |
| ENSG00000164303 | A | mRNA |
| ENSG00000164574 | A | mRNA |
| ENSG00000166090 | A | mRNA |
| ENSG00000166340 | A | mRNA |
| ENSG00000175806 | A | mRNA |
| ENSG00000202058 | A | mRNA |
| ENSG00000204869 | A | mRNA |
| ENSG00000211789 | A | mRNA |
| ENSG00000214264 | A | mRNA |
| ENSG00000215009 | A | mRNA |
| ENSG00000225932 | A | mRNA |
| ENSG00000227259 | A | mRNA |
| ENSG00000228259 | A | mRNA |
| ENSG00000230350 | A | mRNA |
| ENSG00000231305 | A | antisense |
| ENSG00000231964 | A | antisense |
| ENSG00000233549 | A | mRNA |
| ENSG00000236307 | A | mRNA |
| ENSG00000237827 | A | mRNA |
| ENSG00000243896 | A | mRNA |
| ENSG00000249353 | A | mRNA |
| ENSG00000250820 | A | sense_overlapping |
| ENSG00000251368 | A | mRNA |
| ENSG00000252438 | A | mRNA |
| ENSG00000252660 | A | mRNA |
| ENSG00000262566 | A | mRNA |
| ENSG00000265333 | A | mRNA |
| ENSG00000268873 | A | antisense |
| ENSG00000268945 | A | lincRNA |
| ENSG00000271161 | A | mRNA |
| ENSG00000271938 | A | lincRNA |
| ENSG00000278483 | A | mRNA |
| ENSG00000285675 | A | mRNA |
| ENSG00000010244 | B | mRNA |
| ENSG00000025770 | B | mRNA |
| ENSG00000069399 | B | mRNA |
| ENSG00000074964 | B | mRNA |
| ENSG00000100296 | B | mRNA |
| ENSG00000100592 | B | mRNA |
| ENSG00000102010 | B | mRNA |
| ENSG00000102172 | B | mRNA |
| ENSG00000105251 | B | mRNA |
| ENSG00000105697 | B | mRNA |
| ENSG00000105708 | B | mRNA |
| ENSG00000105835 | B | mRNA |
| ENSG00000106028 | B | mRNA |
| ENSG00000106483 | B | mRNA |
| ENSG00000107438 | B | mRNA |
| ENSG00000108179 | B | mRNA |
| ENSG00000109101 | B | mRNA |
| ENSG00000111249 | B | mRNA |
| ENSG00000111341 | B | mRNA |
| ENSG00000115138 | B | mRNA |
| ENSG00000115661 | B | mRNA |
| ENSG00000118200 | B | mRNA |
| ENSG00000121741 | B | mRNA |
| ENSG00000130177 | B | mRNA |
| ENSG00000131652 | B | mRNA |
| ENSG00000132953 | B | mRNA |
| ENSG00000134815 | B | mRNA |
| ENSG00000138785 | B | mRNA |
| ENSG00000140320 | B | mRNA |
| ENSG00000143375 | B | mRNA |
| ENSG00000147041 | B | mRNA |
| ENSG00000147669 | B | mRNA |
| ENSG00000150048 | B | mRNA |
| ENSG00000152229 | B | mRNA |
| ENSG00000157214 | B | mRNA |
| ENSG00000158552 | B | mRNA |
| ENSG00000159885 | B | mRNA |
| ENSG00000164402 | B | mRNA |
| ENSG00000165076 | B | mRNA |
| ENSG00000167637 | B | mRNA |
| ENSG00000168438 | B | mRNA |
| ENSG00000172965 | B | lincRNA |
| ENSG00000173200 | B | mRNA |
| ENSG00000174130 | B | mRNA |
| ENSG00000174939 | B | mRNA |
| ENSG00000176009 | B | mRNA |
| ENSG00000177590 | B | mRNA |
| ENSG00000178440 | B | lincRNA |
| ENSG00000179240 | B | mRNA |
| ENSG00000179520 | B | mRNA |
| ENSG00000181323 | B | mRNA |
| ENSG00000182400 | B | mRNA |
| ENSG00000184140 | B | mRNA |
| ENSG00000184619 | B | mRNA |
| ENSG00000188566 | B | mRNA |
| ENSG00000196634 | B | lincRNA |
| ENSG00000197557 | B | mRNA |
| ENSG00000198863 | B | mRNA |
| ENSG00000199788 | B | mRNA |
| ENSG00000202306 | B | mRNA |
| ENSG00000203288 | B | antisense |
| ENSG00000204711 | B | mRNA |
| ENSG00000206106 | B | mRNA |
| ENSG00000207756 | B | mRNA |
| ENSG00000210107 | B | mRNA |
| ENSG00000216639 | B | mRNA |
| ENSG00000222666 | B | mRNA |
| ENSG00000225511 | B | lincRNA |
| ENSG00000226258 | B | lincRNA |
| ENSG00000226746 | B | antisense |
| ENSG00000226871 | B | antisense |
| ENSG00000227417 | B | mRNA |
| ENSG00000227482 | B | lincRNA |
| ENSG00000227609 | B | mRNA |
| ENSG00000227999 | B | mRNA |
| ENSG00000228221 | B | lincRNA |
| ENSG00000228367 | B | mRNA |
| ENSG00000228420 | B | lincRNA |
| ENSG00000228709 | B | lincRNA |
| ENSG00000229150 | B | mRNA |
| ENSG00000230274 | B | mRNA |
| ENSG00000231390 | B | mRNA |
| ENSG00000232716 | B | mRNA |
| ENSG00000233077 | B | lincRNA |
| ENSG00000233623 | B | mRNA |
| ENSG00000234109 | B | mRNA |
| ENSG00000236445 | B | antisense |
| ENSG00000237172 | B | mRNA |
| ENSG00000240980 | B | lincRNA |
| ENSG00000241926 | B | mRNA |
| ENSG00000244361 | B | mRNA |
| ENSG00000244378 | B | mRNA |
| ENSG00000249372 | B | mRNA |
| ENSG00000250451 | B | antisense |
| ENSG00000250507 | B | mRNA |
| ENSG00000250708 | B | lincRNA |
| ENSG00000252200 | B | mRNA |
| ENSG00000252429 | B | mRNA |
| ENSG00000255670 | B | lincRNA |
| ENSG00000259561 | B | sense_intronic |
| ENSG00000260318 | B | mRNA |
| ENSG00000262884 | B | antisense |
| ENSG00000263574 | B | lincRNA |
| ENSG00000264015 | B | lincRNA |
| ENSG00000264311 | B | mRNA |
| ENSG00000264722 | B | mRNA |
| ENSG00000264739 | B | antisense |
| ENSG00000267137 | B | antisense |
| ENSG00000267214 | B | antisense |
| ENSG00000267666 | B | lincRNA |
| ENSG00000267952 | B | mRNA |
| ENSG00000271269 | B | mRNA |
| ENSG00000271410 | B | mRNA |
| ENSG00000271860 | B | lincRNA |
| ENSG00000272129 | B | lincRNA |
| ENSG00000273024 | B | mRNA |
| ENSG00000275607 | B | mRNA |
| ENSG00000276650 | B | mRNA |
| ENSG00000276717 | B | mRNA |
| ENSG00000278905 | B | TEC |
| ENSG00000286020 | B | mRNA |
| ENSG00000286044 | B | mRNA |
| ENSG00000287180 | B | mRNA |
| ENSG00000287337 | B | mRNA |
| ENSG00000287495 | B | mRNA |
| ENSG00000287884 | B | mRNA |
| ENSG00000288610 | B | mRNA |
| ENSG00000100979 | C | mRNA |
| ENSG00000132623 | C | mRNA |
| ENSG00000143436 | C | mRNA |
| ENSG00000144048 | C | mRNA |
| ENSG00000165338 | C | mRNA |
| ENSG00000167004 | C | mRNA |
| ENSG00000168036 | C | mRNA |
| ENSG00000179921 | C | mRNA |
| ENSG00000189120 | C | mRNA |
| ENSG00000197905 | C | mRNA |
| ENSG00000198931 | C | mRNA |
| ENSG00000213667 | C | mRNA |
| ENSG00000214223 | C | mRNA |
| ENSG00000214552 | C | mRNA |
| ENSG00000216316 | C | mRNA |
| ENSG00000227719 | C | antisense |
| ENSG00000230131 | C | lincRNA |
| ENSG00000230568 | C | mRNA |
| ENSG00000231258 | C | mRNA |
| ENSG00000235449 | C | mRNA |
| ENSG00000235613 | C | mRNA |
| ENSG00000236267 | C | antisense |
| ENSG00000236319 | C | mRNA |
| ENSG00000236905 | C | mRNA |
| ENSG00000237418 | C | mRNA |
| ENSG00000241587 | C | mRNA |
| ENSG00000241657 | C | lincRNA |
| ENSG00000241890 | C | mRNA |
| ENSG00000243250 | C | mRNA |
| ENSG00000251118 | C | mRNA |
| ENSG00000253092 | C | mRNA |
| ENSG00000253413 | C | mRNA |
| ENSG00000257016 | C | mRNA |
| ENSG00000258623 | C | antisense |
| ENSG00000261018 | C | antisense |
| ENSG00000261338 | C | sense_overlapping |
| ENSG00000261594 | C | mRNA |
| ENSG00000264785 | C | antisense |
| ENSG00000265648 | C | mRNA |
| ENSG00000267089 | C | lincRNA |
| ENSG00000268412 | C | mRNA |
| ENSG00000269997 | C | sense_intronic |
| ENSG00000271204 | C | lincRNA |
| ENSG00000279953 | C | TEC |
| ENSG00000287710 | C | mRNA |
| ENSG00000003056 | D | mRNA |
| ENSG00000079950 | D | mRNA |
| ENSG00000086475 | D | mRNA |
| ENSG00000087301 | D | mRNA |
| ENSG00000095587 | D | mRNA |
| ENSG00000100505 | D | mRNA |
| ENSG00000103043 | D | mRNA |
| ENSG00000104938 | D | mRNA |
| ENSG00000105866 | D | mRNA |
| ENSG00000111696 | D | mRNA |
| ENSG00000111752 | D | mRNA |
| ENSG00000115539 | D | mRNA |
| ENSG00000118520 | D | mRNA |
| ENSG00000124159 | D | mRNA |
| ENSG00000125611 | D | mRNA |
| ENSG00000130711 | D | mRNA |
| ENSG00000134058 | D | mRNA |
| ENSG00000134152 | D | mRNA |
| ENSG00000136694 | D | mRNA |
| ENSG00000136944 | D | mRNA |
| ENSG00000137473 | D | mRNA |
| ENSG00000138668 | D | mRNA |
| ENSG00000139160 | D | mRNA |
| ENSG00000139351 | D | mRNA |
| ENSG00000140022 | D | mRNA |
| ENSG00000142182 | D | mRNA |
| ENSG00000144821 | D | mRNA |
| ENSG00000145687 | D | mRNA |
| ENSG00000153006 | D | mRNA |
| ENSG00000153446 | D | mRNA |
| ENSG00000154079 | D | mRNA |
| ENSG00000158716 | D | mRNA |
| ENSG00000160305 | D | mRNA |
| ENSG00000160345 | D | mRNA |
| ENSG00000161328 | D | mRNA |
| ENSG00000162396 | D | mRNA |
| ENSG00000163584 | D | mRNA |
| ENSG00000170409 | D | mRNA |
| ENSG00000170500 | D | mRNA |
| ENSG00000170855 | D | mRNA |
| ENSG00000171094 | D | mRNA |
| ENSG00000171680 | D | mRNA |
| ENSG00000172215 | D | mRNA |
| ENSG00000172954 | D | mRNA |
| ENSG00000177025 | D | mRNA |
| ENSG00000181552 | D | mRNA |
| ENSG00000183688 | D | mRNA |
| ENSG00000184232 | D | mRNA |
| ENSG00000186431 | D | mRNA |
| ENSG00000188227 | D | mRNA |
| ENSG00000188322 | D | mRNA |
| ENSG00000188396 | D | mRNA |
| ENSG00000189377 | D | mRNA |
| ENSG00000203907 | D | mRNA |
| ENSG00000204745 | D | mRNA |
| ENSG00000223779 | D | mRNA |
| ENSG00000225564 | D | lincRNA |
| ENSG00000225813 | D | mRNA |
| ENSG00000226491 | D | mRNA |
| ENSG00000226741 | D | lincRNA |
| ENSG00000226927 | D | lincRNA |
| ENSG00000227949 | D | mRNA |
| ENSG00000230104 | D | mRNA |
| ENSG00000231017 | D | mRNA |
| ENSG00000231395 | D | mRNA |
| ENSG00000231563 | D | antisense |
| ENSG00000232110 | D | antisense |
| ENSG00000234143 | D | mRNA |
| ENSG00000234361 | D | antisense |
| ENSG00000234607 | D | mRNA |
| ENSG00000235910 | D | antisense |
| ENSG00000237167 | D | lincRNA |
| ENSG00000238374 | D | mRNA |
| ENSG00000243883 | D | mRNA |
| ENSG00000248971 | D | mRNA |
| ENSG00000251019 | D | mRNA |
| ENSG00000251165 | D | antisense |
| ENSG00000254206 | D | mRNA |
| ENSG00000254350 | D | lincRNA |
| ENSG00000256223 | D | mRNA |
| ENSG00000256817 | D | mRNA |
| ENSG00000256937 | D | mRNA |
| ENSG00000257757 | D | mRNA |
| ENSG00000260231 | D | antisense |
| ENSG00000260286 | D | mRNA |
| ENSG00000260410 | D | mRNA |
| ENSG00000260740 | D | antisense |
| ENSG00000261098 | D | lincRNA |
| ENSG00000261584 | D | lincRNA |
| ENSG00000261603 | D | mRNA |
| ENSG00000261822 | D | antisense |
| ENSG00000267858 | D | antisense |
| ENSG00000269001 | D | mRNA |
| ENSG00000269482 | D | antisense |
| ENSG00000269680 | D | antisense |
| ENSG00000269699 | D | mRNA |
| ENSG00000270188 | D | mRNA |
| ENSG00000271714 | D | antisense |
| ENSG00000272128 | D | lincRNA |
| ENSG00000272158 | D | antisense |
| ENSG00000272275 | D | lincRNA |
| ENSG00000272305 | D | mRNA |
| ENSG00000273513 | D | mRNA |
| ENSG00000275532 | D | antisense |
| ENSG00000277406 | D | mRNA |
| ENSG00000283156 | D | mRNA |
| ENSG00000286062 | D | mRNA |
| ENSG00000286174 | D | mRNA |
| ENSG00000287330 | D | mRNA |
| ENSG00000287411 | D | mRNA |
| ENSG00000288050 | D | mRNA |
| ENSG00000072518 | E | mRNA |
| ENSG00000084652 | E | mRNA |
| ENSG00000100359 | E | mRNA |
| ENSG00000101115 | E | mRNA |
| ENSG00000112293 | E | mRNA |
| ENSG00000118507 | E | mRNA |
| ENSG00000119866 | E | mRNA |
| ENSG00000128322 | E | mRNA |
| ENSG00000135912 | E | mRNA |
| ENSG00000137656 | E | mRNA |
| ENSG00000158571 | E | mRNA |
| ENSG00000160226 | E | mRNA |
| ENSG00000161955 | E | mRNA |
| ENSG00000164307 | E | mRNA |
| ENSG00000169246 | E | mRNA |
| ENSG00000170832 | E | mRNA |
| ENSG00000174677 | E | mRNA |
| ENSG00000177776 | E | mRNA |
| ENSG00000182774 | E | mRNA |
| ENSG00000197256 | E | mRNA |
| ENSG00000198920 | E | mRNA |
| ENSG00000199756 | E | mRNA |
| ENSG00000199809 | E | mRNA |
| ENSG00000200135 | E | mRNA |
| ENSG00000201448 | E | mRNA |
| ENSG00000201554 | E | mRNA |
| ENSG00000201747 | E | mRNA |
| ENSG00000204681 | E | mRNA |
| ENSG00000206633 | E | mRNA |
| ENSG00000207326 | E | mRNA |
| ENSG00000211930 | E | mRNA |
| ENSG00000214745 | E | mRNA |
| ENSG00000215444 | E | mRNA |
| ENSG00000218350 | E | mRNA |
| ENSG00000218730 | E | mRNA |
| ENSG00000220960 | E | mRNA |
| ENSG00000224733 | E | antisense |
| ENSG00000225777 | E | mRNA |
| ENSG00000228742 | E | lincRNA |
| ENSG00000229002 | E | mRNA |
| ENSG00000229229 | E | antisense |
| ENSG00000230154 | E | mRNA |
| ENSG00000230476 | E | mRNA |
| ENSG00000231332 | E | antisense |
| ENSG00000232434 | E | mRNA |
| ENSG00000233259 | E | mRNA |
| ENSG00000233264 | E | mRNA |
| ENSG00000235358 | E | lincRNA |
| ENSG00000235806 | E | lincRNA |
| ENSG00000240036 | E | mRNA |
| ENSG00000240058 | E | mRNA |
| ENSG00000242028 | E | mRNA |
| ENSG00000248903 | E | mRNA |
| ENSG00000251013 | E | mRNA |
| ENSG00000251497 | E | lincRNA |
| ENSG00000251597 | E | mRNA |
| ENSG00000253654 | E | mRNA |
| ENSG00000254279 | E | mRNA |
| ENSG00000254655 | E | mRNA |
| ENSG00000255434 | E | sense_intronic |
| ENSG00000260194 | E | antisense |
| ENSG00000260859 | E | sense_intronic |
| ENSG00000263427 | E | lincRNA |
| ENSG00000264864 | E | mRNA |
| ENSG00000273979 | E | mRNA |
| ENSG00000276900 | E | antisense |
| ENSG00000277142 | E | lincRNA |
| ENSG00000279649 | E | TEC |
| ENSG00000285639 | E | mRNA |
| ENSG00000104518 | F | mRNA |
| ENSG00000104755 | F | mRNA |
| ENSG00000127780 | F | mRNA |
| ENSG00000128463 | F | mRNA |
| ENSG00000133612 | F | mRNA |
| ENSG00000134056 | F | mRNA |
| ENSG00000134709 | F | mRNA |
| ENSG00000136715 | F | mRNA |
| ENSG00000140497 | F | mRNA |
| ENSG00000144395 | F | mRNA |
| ENSG00000145901 | F | mRNA |
| ENSG00000146376 | F | mRNA |
| ENSG00000149403 | F | mRNA |
| ENSG00000151247 | F | mRNA |
| ENSG00000162947 | F | lincRNA |
| ENSG00000171681 | F | mRNA |
| ENSG00000173585 | F | mRNA |
| ENSG00000184492 | F | mRNA |
| ENSG00000186716 | F | mRNA |
| ENSG00000187747 | F | mRNA |
| ENSG00000198054 | F | mRNA |
| ENSG00000200818 | F | mRNA |
| ENSG00000205822 | F | mRNA |
| ENSG00000206907 | F | mRNA |
| ENSG00000207619 | F | mRNA |
| ENSG00000219294 | F | mRNA |
| ENSG00000223874 | F | lincRNA |
| ENSG00000224247 | F | lincRNA |
| ENSG00000224312 | F | mRNA |
| ENSG00000228423 | F | lincRNA |
| ENSG00000232494 | F | lincRNA |
| ENSG00000253954 | F | mRNA |
| ENSG00000262155 | F | lincRNA |
| ENSG00000264349 | F | mRNA |
| ENSG00000279294 | F | TEC |
| ENSG00000006638 | G | mRNA |
| ENSG00000013725 | G | mRNA |
| ENSG00000039650 | G | mRNA |
| ENSG00000066739 | G | mRNA |
| ENSG00000069696 | G | mRNA |
| ENSG00000075624 | G | mRNA |
| ENSG00000076662 | G | mRNA |
| ENSG00000078070 | G | mRNA |
| ENSG00000085365 | G | mRNA |
| ENSG00000087086 | G | mRNA |
| ENSG00000088727 | G | mRNA |
| ENSG00000095370 | G | mRNA |
| ENSG00000099282 | G | mRNA |
| ENSG00000100058 | G | mRNA |
| ENSG00000104953 | G | mRNA |
| ENSG00000106211 | G | mRNA |
| ENSG00000108306 | G | mRNA |
| ENSG00000108510 | G | mRNA |
| ENSG00000111144 | G | mRNA |
| ENSG00000111261 | G | mRNA |
| ENSG00000112782 | G | mRNA |
| ENSG00000113141 | G | mRNA |
| ENSG00000113532 | G | mRNA |
| ENSG00000114626 | G | mRNA |
| ENSG00000115289 | G | mRNA |
| ENSG00000116035 | G | mRNA |
| ENSG00000117640 | G | mRNA |
| ENSG00000119636 | G | mRNA |
| ENSG00000120053 | G | mRNA |
| ENSG00000120314 | G | mRNA |
| ENSG00000120688 | G | mRNA |
| ENSG00000125821 | G | mRNA |
| ENSG00000127423 | G | mRNA |
| ENSG00000128185 | G | mRNA |
| ENSG00000130529 | G | mRNA |
| ENSG00000130656 | G | mRNA |
| ENSG00000131401 | G | mRNA |
| ENSG00000132746 | G | mRNA |
| ENSG00000132792 | G | mRNA |
| ENSG00000132801 | G | mRNA |
| ENSG00000134864 | G | mRNA |
| ENSG00000139187 | G | mRNA |
| ENSG00000139970 | G | mRNA |
| ENSG00000140297 | G | mRNA |
| ENSG00000142192 | G | mRNA |
| ENSG00000142556 | G | mRNA |
| ENSG00000143067 | G | mRNA |
| ENSG00000150750 | G | mRNA |
| ENSG00000150773 | G | mRNA |
| ENSG00000151090 | G | mRNA |
| ENSG00000151552 | G | mRNA |
| ENSG00000152952 | G | mRNA |
| ENSG00000159761 | G | mRNA |
| ENSG00000159899 | G | mRNA |
| ENSG00000160469 | G | mRNA |
| ENSG00000161265 | G | mRNA |
| ENSG00000162068 | G | mRNA |
| ENSG00000163534 | G | mRNA |
| ENSG00000164638 | G | mRNA |
| ENSG00000164972 | G | mRNA |
| ENSG00000165059 | G | mRNA |
| ENSG00000165973 | G | mRNA |
| ENSG00000166068 | G | mRNA |
| ENSG00000166313 | G | mRNA |
| ENSG00000166780 | G | mRNA |
| ENSG00000166787 | G | mRNA |
| ENSG00000167705 | G | mRNA |
| ENSG00000169093 | G | mRNA |
| ENSG00000170315 | G | mRNA |
| ENSG00000171241 | G | mRNA |
| ENSG00000173221 | G | mRNA |
| ENSG00000173674 | G | mRNA |
| ENSG00000174353 | G | mRNA |
| ENSG00000176049 | G | mRNA |
| ENSG00000178082 | G | mRNA |
| ENSG00000182310 | G | processed_transcript |
| ENSG00000182327 | G | mRNA |
| ENSG00000183305 | G | mRNA |
| ENSG00000184831 | G | mRNA |
| ENSG00000184995 | G | mRNA |
| ENSG00000185163 | G | mRNA |
| ENSG00000196090 | G | mRNA |
| ENSG00000196131 | G | mRNA |
| ENSG00000197587 | G | mRNA |
| ENSG00000198074 | G | mRNA |
| ENSG00000198673 | G | mRNA |
| ENSG00000199646 | G | mRNA |
| ENSG00000201379 | G | mRNA |
| ENSG00000202137 | G | mRNA |
| ENSG00000203808 | G | antisense |
| ENSG00000204887 | G | mRNA |
| ENSG00000205989 | G | mRNA |
| ENSG00000206762 | G | mRNA |
| ENSG00000212932 | G | mRNA |
| ENSG00000213421 | G | mRNA |
| ENSG00000216906 | G | mRNA |
| ENSG00000223619 | G | mRNA |
| ENSG00000226207 | G | lincRNA |
| ENSG00000226899 | G | antisense |
| ENSG00000227454 | G | mRNA |
| ENSG00000228750 | G | lincRNA |
| ENSG00000230691 | G | mRNA |
| ENSG00000230832 | G | mRNA |
| ENSG00000231053 | G | mRNA |
| ENSG00000231210 | G | lincRNA |
| ENSG00000232174 | G | mRNA |
| ENSG00000233609 | G | mRNA |
| ENSG00000236187 | G | mRNA |
| ENSG00000236484 | G | mRNA |
| ENSG00000240370 | G | mRNA |
| ENSG00000244384 | G | mRNA |
| ENSG00000248221 | G | sense_intronic |
| ENSG00000249771 | G | lincRNA |
| ENSG00000250305 | G | mRNA |
| ENSG00000260232 | G | lincRNA |
| ENSG00000265078 | G | mRNA |
| ENSG00000267547 | G | lincRNA |
| ENSG00000267872 | G | lincRNA |
| ENSG00000276390 | G | sense_intronic |
| ENSG00000277149 | G | mRNA |
| ENSG00000277636 | G | mRNA |
| ENSG00000278616 | G | mRNA |
| ENSG00000287624 | G | mRNA |
| ENSG00000012048 | H | mRNA |
| ENSG00000065923 | H | mRNA |
| ENSG00000090339 | H | mRNA |
| ENSG00000104371 | H | mRNA |
| ENSG00000108469 | H | mRNA |
| ENSG00000109686 | H | mRNA |
| ENSG00000114098 | H | mRNA |
| ENSG00000115593 | H | mRNA |
| ENSG00000121486 | H | mRNA |
| ENSG00000127125 | H | mRNA |
| ENSG00000132975 | H | mRNA |
| ENSG00000135655 | H | mRNA |
| ENSG00000154146 | H | mRNA |
| ENSG00000162601 | H | mRNA |
| ENSG00000167114 | H | mRNA |
| ENSG00000173141 | H | mRNA |
| ENSG00000175054 | H | mRNA |
| ENSG00000175518 | H | mRNA |
| ENSG00000184281 | H | mRNA |
| ENSG00000185989 | H | mRNA |
| ENSG00000188582 | H | mRNA |
| ENSG00000197536 | H | mRNA |
| ENSG00000206573 | H | antisense |
| ENSG00000207296 | H | mRNA |
| ENSG00000212440 | H | mRNA |
| ENSG00000212807 | H | mRNA |
| ENSG00000213140 | H | mRNA |
| ENSG00000221946 | H | mRNA |
| ENSG00000223754 | H | antisense |
| ENSG00000226471 | H | antisense |
| ENSG00000231231 | H | lincRNA |
| ENSG00000232978 | H | antisense |
| ENSG00000233893 | H | antisense |
| ENSG00000234753 | H | antisense |
| ENSG00000235162 | H | mRNA |
| ENSG00000236491 | H | mRNA |
| ENSG00000236494 | H | lincRNA |
| ENSG00000237491 | H | lincRNA |
| ENSG00000240254 | H | antisense |
| ENSG00000242071 | H | mRNA |
| ENSG00000243101 | H | mRNA |
| ENSG00000251008 | H | mRNA |
| ENSG00000253779 | H | mRNA |
| ENSG00000254612 | H | mRNA |
| ENSG00000255815 | H | mRNA |
| ENSG00000256427 | H | lincRNA |
| ENSG00000256681 | H | mRNA |
| ENSG00000257084 | H | lincRNA |
| ENSG00000262880 | H | processed_transcript |
| ENSG00000264772 | H | processed_transcript |
| ENSG00000266992 | H | mRNA |
| ENSG00000267057 | H | lincRNA |
| ENSG00000268058 | H | mRNA |
| ENSG00000269956 | H | antisense |
| ENSG00000270654 | H | mRNA |
| ENSG00000270909 | H | mRNA |
| ENSG00000271129 | H | mRNA |
| ENSG00000271216 | H | lincRNA |
| ENSG00000271524 | H | mRNA |
| ENSG00000271584 | H | lincRNA |
| ENSG00000273769 | H | processed_transcript |
| ENSG00000274390 | H | mRNA |
| ENSG00000274923 | H | mRNA |
| ENSG00000277311 | H | mRNA |
| ENSG00000280274 | H | TEC |
| ENSG00000283095 | H | lincRNA |
| ENSG00000284594 | H | mRNA |
| ENSG00000287284 | H | mRNA |
| ENSG00000287312 | H | mRNA |
| ENSG00000287378 | H | mRNA |
| ENSG00000287803 | H | mRNA |
| ENSG00000001629 | I | mRNA |
| ENSG00000015520 | I | mRNA |
| ENSG00000025039 | I | mRNA |
| ENSG00000079785 | I | mRNA |
| ENSG00000080007 | I | mRNA |
| ENSG00000106615 | I | mRNA |
| ENSG00000118785 | I | mRNA |
| ENSG00000120725 | I | mRNA |
| ENSG00000124813 | I | mRNA |
| ENSG00000125538 | I | mRNA |
| ENSG00000126787 | I | mRNA |
| ENSG00000130244 | I | mRNA |
| ENSG00000131634 | I | mRNA |
| ENSG00000133706 | I | mRNA |
| ENSG00000135297 | I | mRNA |
| ENSG00000135968 | I | mRNA |
| ENSG00000137073 | I | mRNA |
| ENSG00000139266 | I | mRNA |
| ENSG00000140718 | I | mRNA |
| ENSG00000140743 | I | mRNA |
| ENSG00000151348 | I | mRNA |
| ENSG00000151576 | I | mRNA |
| ENSG00000152254 | I | mRNA |
| ENSG00000155096 | I | mRNA |
| ENSG00000157045 | I | mRNA |
| ENSG00000157999 | I | mRNA |
| ENSG00000159592 | I | mRNA |
| ENSG00000164828 | I | mRNA |
| ENSG00000166436 | I | mRNA |
| ENSG00000166825 | I | mRNA |
| ENSG00000172717 | I | mRNA |
| ENSG00000174469 | I | mRNA |
| ENSG00000175782 | I | mRNA |
| ENSG00000176761 | I | mRNA |
| ENSG00000181513 | I | mRNA |
| ENSG00000182749 | I | mRNA |
| ENSG00000183092 | I | mRNA |
| ENSG00000183199 | I | mRNA |
| ENSG00000185033 | I | mRNA |
| ENSG00000186481 | I | mRNA |
| ENSG00000187951 | I | mRNA |
| ENSG00000188997 | I | mRNA |
| ENSG00000196335 | I | mRNA |
| ENSG00000196597 | I | mRNA |
| ENSG00000198821 | I | mRNA |
| ENSG00000198851 | I | mRNA |
| ENSG00000205336 | I | mRNA |
| ENSG00000205611 | I | lincRNA |
| ENSG00000215296 | I | mRNA |
| ENSG00000220739 | I | mRNA |
| ENSG00000224743 | I | antisense |
| ENSG00000225133 | I | mRNA |
| ENSG00000227006 | I | antisense |
| ENSG00000227400 | I | sense_overlapping |
| ENSG00000228135 | I | antisense |
| ENSG00000228536 | I | lincRNA |
| ENSG00000229912 | I | lincRNA |
| ENSG00000230162 | I | mRNA |
| ENSG00000230732 | I | sense_intronic |
| ENSG00000230951 | I | mRNA |
| ENSG00000231445 | I | mRNA |
| ENSG00000232283 | I | antisense |
| ENSG00000233487 | I | mRNA |
| ENSG00000233521 | I | lincRNA |
| ENSG00000234638 | I | antisense |
| ENSG00000235314 | I | lincRNA |
| ENSG00000235429 | I | mRNA |
| ENSG00000235501 | I | antisense |
| ENSG00000236088 | I | processed_transcript |
| ENSG00000236204 | I | lincRNA |
| ENSG00000236939 | I | antisense |
| ENSG00000237540 | I | mRNA |
| ENSG00000237614 | I | lincRNA |
| ENSG00000240024 | I | mRNA |
| ENSG00000241400 | I | mRNA |
| ENSG00000243323 | I | mRNA |
| ENSG00000244264 | I | mRNA |
| ENSG00000244687 | I | mRNA |
| ENSG00000246526 | I | lincRNA |
| ENSG00000247572 | I | antisense |
| ENSG00000247708 | I | antisense |
| ENSG00000247982 | I | lincRNA |
| ENSG00000249944 | I | mRNA |
| ENSG00000250158 | I | lincRNA |
| ENSG00000250252 | I | lincRNA |
| ENSG00000250312 | I | mRNA |
| ENSG00000250815 | I | mRNA |
| ENSG00000251148 | I | antisense |
| ENSG00000253032 | I | mRNA |
| ENSG00000253618 | I | antisense |
| ENSG00000253891 | I | lincRNA |
| ENSG00000254251 | I | antisense |
| ENSG00000254326 | I | mRNA |
| ENSG00000254634 | I | mRNA |
| ENSG00000255185 | I | mRNA |
| ENSG00000255384 | I | lincRNA |
| ENSG00000255389 | I | antisense |
| ENSG00000255837 | I | mRNA |
| ENSG00000256969 | I | lincRNA |
| ENSG00000257127 | I | mRNA |
| ENSG00000257337 | I | antisense |
| ENSG00000257607 | I | antisense |
| ENSG00000258791 | I | lincRNA |
| ENSG00000259043 | I | mRNA |
| ENSG00000259201 | I | antisense |
| ENSG00000259299 | I | mRNA |
| ENSG00000259541 | I | antisense |
| ENSG00000259607 | I | antisense |
| ENSG00000259821 | I | lincRNA |
| ENSG00000260084 | I | antisense |
| ENSG00000260230 | I | mRNA |
| ENSG00000260865 | I | mRNA |
| ENSG00000260870 | I | mRNA |
| ENSG00000260891 | I | antisense |
| ENSG00000260969 | I | antisense |
| ENSG00000261092 | I | lincRNA |
| ENSG00000261739 | I | mRNA |
| ENSG00000261745 | I | sense_overlapping |
| ENSG00000263941 | I | mRNA |
| ENSG00000264026 | I | lincRNA |
| ENSG00000264145 | I | mRNA |
| ENSG00000265494 | I | lincRNA |
| ENSG00000266188 | I | mRNA |
| ENSG00000266497 | I | mRNA |
| ENSG00000267065 | I | lincRNA |
| ENSG00000267199 | I | antisense |
| ENSG00000267253 | I | mRNA |
| ENSG00000267508 | I | mRNA |
| ENSG00000269385 | I | mRNA |
| ENSG00000269473 | I | lincRNA |
| ENSG00000269506 | I | antisense |
| ENSG00000270017 | I | lincRNA |
| ENSG00000270039 | I | lincRNA |
| ENSG00000270890 | I | mRNA |
| ENSG00000270945 | I | mRNA |
| ENSG00000271553 | I | lincRNA |
| ENSG00000271605 | I | mRNA |
| ENSG00000272078 | I | antisense |
| ENSG00000272382 | I | lincRNA |
| ENSG00000272446 | I | processed_transcript |
| ENSG00000272498 | I | antisense |
| ENSG00000272545 | I | lincRNA |
| ENSG00000273474 | I | antisense |
| ENSG00000273680 | I | antisense |
| ENSG00000274070 | I | mRNA |
| ENSG00000274322 | I | mRNA |
| ENSG00000275393 | I | sense_intronic |
| ENSG00000275426 | I | sense_intronic |
| ENSG00000275451 | I | mRNA |
| ENSG00000276710 | I | mRNA |
| ENSG00000277440 | I | sense_intronic |
| ENSG00000277496 | I | antisense |
| ENSG00000277969 | I | lincRNA |
| ENSG00000279466 | I | TEC |
| ENSG00000279529 | I | antisense |
| ENSG00000279887 | I | TEC |
| ENSG00000279897 | I | antisense |
| ENSG00000279926 | I | TEC |
| ENSG00000280067 | I | TEC |
| ENSG00000280115 | I | TEC |
| ENSG00000280623 | I | lincRNA |
| ENSG00000281167 | I | lincRNA |
| ENSG00000283914 | I | mRNA |
| ENSG00000283972 | I | mRNA |
| ENSG00000284669 | I | mRNA |
| ENSG00000284882 | I | mRNA |
| ENSG00000285287 | I | mRNA |
| ENSG00000285728 | I | mRNA |
| ENSG00000286092 | I | mRNA |
| ENSG00000286389 | I | mRNA |
| ENSG00000286512 | I | mRNA |
| ENSG00000286737 | I | mRNA |
| ENSG00000286822 | I | mRNA |
| ENSG00000286894 | I | mRNA |
| ENSG00000286970 | I | mRNA |
| ENSG00000287149 | I | mRNA |
| ENSG00000287262 | I | mRNA |
| ENSG00000287338 | I | mRNA |
| ENSG00000287386 | I | mRNA |
| ENSG00000287608 | I | mRNA |
| ENSG00000287978 | I | mRNA |
| ENSG00000288139 | I | mRNA |
| ENSG00000050327 | J | mRNA |
| ENSG00000060971 | J | mRNA |
| ENSG00000104731 | J | mRNA |
| ENSG00000120694 | J | mRNA |
| ENSG00000123407 | J | mRNA |
| ENSG00000146833 | J | mRNA |
| ENSG00000163064 | J | mRNA |
| ENSG00000167566 | J | mRNA |
| ENSG00000174106 | J | mRNA |
| ENSG00000175535 | J | mRNA |
| ENSG00000178803 | J | antisense |
| ENSG00000178917 | J | mRNA |
| ENSG00000188818 | J | mRNA |
| ENSG00000196437 | J | mRNA |
| ENSG00000200728 | J | mRNA |
| ENSG00000212124 | J | mRNA |
| ENSG00000212321 | J | mRNA |
| ENSG00000221545 | J | mRNA |
| ENSG00000222268 | J | mRNA |
| ENSG00000227403 | J | lincRNA |
| ENSG00000229462 | J | mRNA |
| ENSG00000230126 | J | antisense |
| ENSG00000230778 | J | mRNA |
| ENSG00000232871 | J | mRNA |
| ENSG00000233223 | J | antisense |
| ENSG00000233291 | J | mRNA |
| ENSG00000233622 | J | mRNA |
| ENSG00000233979 | J | mRNA |
| ENSG00000234005 | J | mRNA |
| ENSG00000234752 | J | lincRNA |
| ENSG00000238029 | J | mRNA |
| ENSG00000239839 | J | mRNA |
| ENSG00000240861 | J | mRNA |
| ENSG00000241490 | J | antisense |
| ENSG00000241868 | J | mRNA |
| ENSG00000241899 | J | mRNA |
| ENSG00000242107 | J | lincRNA |
| ENSG00000250929 | J | antisense |
| ENSG00000251078 | J | mRNA |
| ENSG00000251550 | J | mRNA |
| ENSG00000253659 | J | lincRNA |
| ENSG00000254469 | J | mRNA |
| ENSG00000254488 | J | lincRNA |
| ENSG00000257687 | J | mRNA |
| ENSG00000257720 | J | mRNA |
| ENSG00000259278 | J | lincRNA |
| ENSG00000260742 | J | antisense |
| ENSG00000260889 | J | mRNA |
| ENSG00000261823 | J | lincRNA |
| ENSG00000263159 | J | antisense |
| ENSG00000264313 | J | mRNA |
| ENSG00000265060 | J | mRNA |
| ENSG00000269444 | J | sense_overlapping |
| ENSG00000270367 | J | mRNA |
| ENSG00000270424 | J | mRNA |
| ENSG00000271287 | J | mRNA |
| ENSG00000271984 | J | antisense |
| ENSG00000272337 | J | mRNA |
| ENSG00000272736 | J | antisense |
| ENSG00000275680 | J | mRNA |
| ENSG00000277488 | J | mRNA |
| ENSG00000283445 | J | mRNA |
| ENSG00000285253 | J | mRNA |
| ENSG00000286016 | J | mRNA |
| ENSG00000286466 | J | mRNA |
| ENSG00000286627 | J | mRNA |
| ENSG00000286633 | J | mRNA |
| ENSG00000286642 | J | mRNA |
| ENSG00000287627 | J | mRNA |
| ENSG00000287628 | J | mRNA |

**Supplementary Table 2.** Inference of infiltrating cells from gene expression profiles.

| Cell types | HC | | | FrO | | | Fr/TBI | | | P value (One-way ANOVA) |
| --- | --- | --- | --- | --- | --- | --- | --- | --- | --- | --- |
|  |  |  |  |  |  |  |  |  |  |  |
| Microvascular endothelial cells | 0.0000 | 0.0000 | 0.0000 | 0.0042 | 0.0013 | 0.0003 | 0.0056 | 0.0042 | 0.0053 | 0.0069 |
| B-cells | 0.0258 | 0.0407 | 0.0278 | 0.0000 | 0.0153 | 0.0209 | 0.0053 | 0.0000 | 0.0063 | 0.0145 |
| naive B-cells | 0.0193 | 0.0206 | 0.0204 | 0.0000 | 0.0089 | 0.0128 | 0.0090 | 0.0000 | 0.0064 | 0.0153 |
| Basophils | 0.0339 | 0.0211 | 0.0580 | 0.0000 | 0.0000 | 0.0047 | 0.0043 | 0.0125 | 0.0000 | 0.0162 |
| CD8+ effector memory T-cells | 0.0000 | 0.0000 | 0.0000 | 0.0000 | 0.0000 | 0.0000 | 0.0000 | 0.0000 | 0.0000 | 0.0354 |
| Sebocytes | 0.0000 | 0.0000 | 0.0000 | 0.0000 | 0.0000 | 0.0000 | 0.0000 | 0.0000 | 0.0000 | 0.1009 |
| Type 2 T-helper cells | 0.0613 | 0.0204 | 0.0385 | 0.0000 | 0.0321 | 0.0287 | 0.0154 | 0.0000 | 0.0079 | 0.1232 |
| Type 1 T-helper cells | 0.0000 | 0.0000 | 0.0000 | 0.0000 | 0.0000 | 0.0000 | 0.0000 | 0.0006 | 0.0012 | 0.1251 |
| Osteoblast | 0.0000 | 0.0000 | 0.0000 | 0.0013 | 0.0000 | 0.0028 | 0.0000 | 0.0000 | 0.0000 | 0.1377 |
| Immature dendritic cells | 0.0021 | 0.0000 | 0.0000 | 0.0169 | 0.0109 | 0.0177 | 0.0210 | 0.0321 | 0.0000 | 0.1458 |
| Monocytes | 0.0000 | 0.0000 | 0.0000 | 0.0000 | 0.0000 | 0.0000 | 0.0000 | 0.0000 | 0.0000 | 0.1465 |
| Hematopoietic stem cells | 0.0132 | 0.0000 | 0.0164 | 0.0607 | 0.0178 | 0.0143 | 0.0318 | 0.0638 | 0.0347 | 0.1666 |
| Erythrocytes | 0.0000 | 0.0000 | 0.0000 | 0.0000 | 0.0000 | 0.0000 | 0.0000 | 0.0000 | 0.0000 | 0.1816 |
| Skeletal muscle | 0.0000 | 0.0000 | 0.0000 | 0.0000 | 0.0000 | 0.0000 | 0.0000 | 0.0000 | 0.0000 | 0.1926 |
| NK cells | 0.0000 | 0.0000 | 0.0000 | 0.0000 | 0.0000 | 0.0000 | 0.0000 | 0.0000 | 0.0000 | 0.2000 |
| Astrocytes | 0.0000 | 0.0000 | 0.0000 | 0.0000 | 0.0000 | 0.0000 | 0.0000 | 0.0000 | 0.0000 | 0.3300 |
| Megakaryocytes | 0.0000 | 0.0000 | 0.0000 | 0.0027 | 0.0000 | 0.0003 | 0.0000 | 0.0000 | 0.0000 | 0.3344 |
| CD8+ naive T-cells | 0.0000 | 0.0000 | 0.0000 | 0.0000 | 0.0000 | 0.0000 | 0.0000 | 0.0000 | 0.0000 | 0.3450 |
| Fibroblasts | 0.0049 | 0.0000 | 0.0000 | 0.0189 | 0.0000 | 0.0035 | 0.0000 | 0.0000 | 0.0000 | 0.3489 |
| Stroma Score | 0.0025 | 0.0000 | 0.0000 | 0.0101 | 0.0002 | 0.0017 | 0.0000 | 0.0000 | 0.0007 | 0.3567 |
| Megakaryocyte-erythroid progenitors | 0.0042 | 0.0000 | 0.0000 | 0.0026 | 0.0060 | 0.0000 | 0.0000 | 0.0001 | 0.0000 | 0.3625 |
| Common lymphoid progenitors | 0.0198 | 0.0248 | 0.0101 | 0.0000 | 0.0128 | 0.0060 | 0.0025 | 0.0303 | 0.0125 | 0.3712 |
| Conventional dendritic cells | 0.0000 | 0.0000 | 0.0000 | 0.0000 | 0.0000 | 0.0013 | 0.0024 | 0.0000 | 0.0007 | 0.3727 |
| Mesangial cells | 0.0000 | 0.0000 | 0.0000 | 0.0000 | 0.0000 | 0.0000 | 0.0000 | 0.0000 | 0.0000 | 0.3996 |
| Pericytes | 0.0000 | 0.0141 | 0.0293 | 0.0311 | 0.0040 | 0.0000 | 0.0000 | 0.0000 | 0.0000 | 0.4036 |
| Gamma delta T-cells | 0.0000 | 0.0000 | 0.0000 | 0.0000 | 0.0000 | 0.0000 | 0.0000 | 0.0000 | 0.0000 | 0.4175 |
| CD8+ T-cells | 0.0000 | 0.0000 | 0.0000 | 0.0000 | 0.0000 | 0.0023 | 0.0000 | 0.0000 | 0.0000 | 0.4219 |
| CD8+ central memory T-cells | 0.0000 | 0.0000 | 0.0000 | 0.0000 | 0.0000 | 0.0011 | 0.0000 | 0.0000 | 0.0000 | 0.4219 |
| Keratinocytes | 0.0000 | 0.0000 | 0.0000 | 0.0000 | 0.0000 | 0.0000 | 0.0000 | 0.0000 | 0.0000 | 0.4219 |
| Lymphatic endothelial cells | 0.0000 | 0.0000 | 0.0000 | 0.0000 | 0.0000 | 0.0002 | 0.0000 | 0.0000 | 0.0000 | 0.4219 |
| Memory B-cells | 0.0000 | 0.0018 | 0.0000 | 0.0000 | 0.0000 | 0.0000 | 0.0000 | 0.0000 | 0.0000 | 0.4219 |
| Neutrophils | 0.0000 | 0.0000 | 0.0000 | 0.0000 | 0.0000 | 0.0000 | 0.0004 | 0.0000 | 0.0000 | 0.4219 |
| Plasmacytoid dendritic cells | 0.0000 | 0.0000 | 0.0000 | 0.0000 | 0.0000 | 0.0003 | 0.0000 | 0.0000 | 0.0000 | 0.4219 |
| Preadipocytes | 0.0000 | 0.0000 | 0.0000 | 0.0000 | 0.0000 | 0.0000 | 0.0000 | 0.0000 | 0.0042 | 0.4219 |
| Multipotent rogenitors | 0.0000 | 0.0000 | 0.0000 | 0.0000 | 0.0000 | 0.0000 | 0.0000 | 0.0000 | 0.0000 | 0.4458 |
| Mesenchymal stem cells | 0.0464 | 0.0026 | 0.0734 | 0.0000 | 0.0170 | 0.0310 | 0.0299 | 0.0201 | 0.0273 | 0.4507 |
| Macrophages | 0.0000 | 0.0000 | 0.0000 | 0.0000 | 0.0000 | 0.0001 | 0.0000 | 0.0000 | 0.0006 | 0.4667 |
| Endothelial cells | 0.0000 | 0.0000 | 0.0000 | 0.0012 | 0.0005 | 0.0000 | 0.0000 | 0.0000 | 0.0014 | 0.4910 |
| Common myeloid progenitors | 0.0000 | 0.0000 | 0.0000 | 0.0043 | 0.0035 | 0.0000 | 0.0100 | 0.0000 | 0.0002 | 0.5101 |
| Platelets | 0.0000 | 0.0000 | 0.0000 | 0.0007 | 0.0009 | 0.0000 | 0.0000 | 0.0000 | 0.0019 | 0.5206 |
| Regulatory T-cells | 0.0000 | 0.0090 | 0.0000 | 0.0000 | 0.0000 | 0.0006 | 0.0000 | 0.0019 | 0.0003 | 0.5322 |
| Dendritic cells | 0.0000 | 0.0000 | 0.0000 | 0.0000 | 0.0000 | 0.0005 | 0.0000 | 0.0000 | 0.0014 | 0.5328 |
| Epithelial cells | 0.0000 | 0.0000 | 0.0000 | 0.0000 | 0.0000 | 0.0003 | 0.0001 | 0.0000 | 0.0002 | 0.5407 |
| Activated dendritic cells | 0.0035 | 0.0456 | 0.0162 | 0.0000 | 0.0004 | 0.0020 | 0.0000 | 0.0011 | 0.0763 | 0.5407 |
| Hepatocytes | 0.0000 | 0.0000 | 0.0000 | 0.0000 | 0.0000 | 0.0000 | 0.0000 | 0.0000 | 0.0000 | 0.5455 |
| Eosinophils | 0.0000 | 0.0000 | 0.0000 | 0.0000 | 0.0000 | 0.0015 | 0.0000 | 0.0000 | 0.0034 | 0.5637 |
| Adipocytes | 0.0000 | 0.0000 | 0.0000 | 0.0000 | 0.0000 | 0.0000 | 0.0000 | 0.0000 | 0.0000 | 0.6026 |
| CD4+ naive T-cells | 0.0000 | 0.0000 | 0.0000 | 0.0000 | 0.0000 | 0.0000 | 0.0000 | 0.0000 | 0.0000 | 0.6094 |
| CD4+ effector memory T-cells | 0.0000 | 0.0000 | 0.0000 | 0.0000 | 0.0000 | 0.0050 | 0.0000 | 0.0000 | 0.0071 | 0.6154 |
| pro B-cells | 0.0028 | 0.0000 | 0.0000 | 0.0000 | 0.0020 | 0.0000 | 0.0000 | 0.0000 | 0.0000 | 0.6154 |
| Neurons | 0.0001 | 0.0000 | 0.0004 | 0.0000 | 0.0001 | 0.0002 | 0.0001 | 0.0000 | 0.0001 | 0.6351 |
| Macrophages M2 | 0.0000 | 0.0002 | 0.0014 | 0.0050 | 0.0000 | 0.0007 | 0.0004 | 0.0020 | 0.0009 | 0.6391 |
| CD4+ T-cells | 0.0000 | 0.0057 | 0.0000 | 0.0000 | 0.0000 | 0.0098 | 0.0000 | 0.0000 | 0.0010 | 0.6579 |
| Smooth muscle | 0.0203 | 0.0924 | 0.0427 | 0.0000 | 0.0983 | 0.1026 | 0.0683 | 0.0997 | 0.0778 | 0.6805 |
| Macrophages M1 | 0.0000 | 0.0000 | 0.0000 | 0.0000 | 0.0000 | 0.0000 | 0.0000 | 0.0000 | 0.0000 | 0.7057 |
| CD4+ memory T-cells | 0.0004 | 0.0072 | 0.0000 | 0.0000 | 0.0024 | 0.0062 | 0.0000 | 0.0000 | 0.0026 | 0.7111 |
| Myocytes | 0.0014 | 0.0022 | 0.0010 | 0.0000 | 0.0000 | 0.0053 | 0.0089 | 0.0000 | 0.0018 | 0.7184 |
| Chondrocytes | 0.0000 | 0.0000 | 0.0000 | 0.0000 | 0.0000 | 0.0000 | 0.0000 | 0.0000 | 0.0000 | 0.7377 |
| Natural killer T-cells | 0.0000 | 0.0034 | 0.0000 | 0.0000 | 0.0015 | 0.0046 | 0.0021 | 0.0000 | 0.0060 | 0.7435 |
| Plasma cells | 0.0019 | 0.0011 | 0.0000 | 0.0013 | 0.0023 | 0.0000 | 0.0006 | 0.0060 | 0.0000 | 0.7523 |
| Class-switched memory B-cells | 0.0024 | 0.0070 | 0.0038 | 0.0000 | 0.0112 | 0.0000 | 0.0054 | 0.0000 | 0.0000 | 0.7566 |
| Mast cells | 0.0007 | 0.0006 | 0.0001 | 0.0000 | 0.0000 | 0.0015 | 0.0000 | 0.0006 | 0.0000 | 0.7835 |
| CD4+ central memory T-cells | 0.0000 | 0.0000 | 0.0000 | 0.0000 | 0.0000 | 0.0000 | 0.0000 | 0.0000 | 0.0000 | 0.9333 |
| Melanocytes | 0.0002 | 0.0001 | 0.0007 | 0.0000 | 0.0001 | 0.0007 | 0.0001 | 0.0001 | 0.0009 | 0.9718 |

**Supplementary Table 3.** lncRNA-mRNA correlation analysis.

| Var1 | Var2 | R value | Gene |
| --- | --- | --- | --- |
| ENSG00000240980 | ENSG00000264145 | -0.9299 | AC239859.3 |
| ENSG00000196634 | ENSG00000215296 | -0.9270 | TMCO5B |
| ENSG00000255670 | ENSG00000286894 | -0.9172 | AC009141.1 |
| ENSG00000240980 | ENSG00000277311 | -0.9171 | AC012354.7 |
| ENSG00000255670 | ENSG00000187951 | -0.9046 | AC091057.1 |
| ENSG00000240980 | ENSG00000287312 | -0.9045 | AC127526.4 |
| ENSG00000240980 | ENSG00000287378 | -0.9045 | AC129803.1 |
| ENSG00000255670 | ENSG00000284594 | -0.8894 | MIR7847 |
| ENSG00000278905 | ENSG00000286822 | -0.8875 | AC112504.3 |
| ENSG00000255670 | ENSG00000183092 | -0.8854 | BEGAIN |
| ENSG00000196634 | ENSG00000250312 | -0.8778 | ZNF718 |
| ENSG00000255670 | ENSG00000259299 | -0.8643 | AC061965.2 |
| ENSG00000278905 | ENSG00000111752 | -0.8587 | PHC1 |
| ENSG00000196634 | ENSG00000140718 | -0.8546 | FTO |
| ENSG00000196634 | ENSG00000133706 | -0.8507 | LARS1 |
| ENSG00000196634 | ENSG00000286627 | -0.8507 | AP005900.1 |
| ENSG00000196634 | ENSG00000286737 | -0.8497 | AC092168.3 |
| ENSG00000240980 | ENSG00000236491 | -0.8429 | AC234771.4 |
| ENSG00000196634 | ENSG00000151348 | -0.8367 | EXT2 |
| ENSG00000196634 | ENSG00000286512 | -0.8360 | AL031768.2 |
| ENSG00000240980 | ENSG00000186481 | -0.8346 | ANKRD20A5P |
| ENSG00000196634 | ENSG00000198821 | -0.8292 | CD247 |
| ENSG00000196634 | ENSG00000244687 | -0.8278 | UBE2V1 |
| ENSG00000278905 | ENSG00000160305 | -0.8238 | DIP2A |
| ENSG00000255670 | ENSG00000271605 | -0.8235 | MILR1 |
| ENSG00000255670 | ENSG00000266188 | -0.8217 | AC099677.6 |
| ENSG00000240980 | ENSG00000274390 | -0.8178 | MIR6885 |
| ENSG00000196634 | ENSG00000205336 | -0.8132 | ADGRG1 |
| ENSG00000278905 | ENSG00000287284 | -0.8083 | AC130814.1 |
| ENSG00000278905 | ENSG00000283156 | -0.8074 | AC068620.3 |
| ENSG00000240980 | ENSG00000184281 | -0.8071 | TSSC4 |
| ENSG00000240980 | ENSG00000127125 | -0.8049 | PPCS |
| ENSG00000255670 | ENSG00000241899 | -0.8032 | TPT1P3 |
| ENSG00000278905 | ENSG00000273513 | -0.8004 | TBC1D3K |
| ENSG00000240980 | ENSG00000270909 | -0.7998 | CR788268.1 |
| ENSG00000255670 | ENSG00000238029 | -0.7920 | RALBP1P2 |
| ENSG00000255670 | ENSG00000174469 | -0.7831 | CNTNAP2 |
| ENSG00000278905 | ENSG00000175782 | -0.7767 | SLC35E3 |
| ENSG00000240980 | ENSG00000274923 | -0.7764 | AC005296.1 |
| ENSG00000278905 | ENSG00000185033 | -0.7761 | SEMA4B |
| ENSG00000240980 | ENSG00000124159 | -0.7720 | MATN4 |
| ENSG00000240980 | ENSG00000118520 | -0.7716 | ARG1 |
| ENSG00000196634 | ENSG00000237540 | -0.7708 | RPL36AP36 |
| ENSG00000278905 | ENSG00000003056 | -0.7698 | M6PR |
| ENSG00000240980 | ENSG00000271129 | -0.7697 | AC009027.1 |
| ENSG00000196634 | ENSG00000288139 | -0.7692 | AL031297.1 |
| ENSG00000255670 | ENSG00000115539 | -0.7655 | PDCL3 |
| ENSG00000255670 | ENSG00000163584 | -0.7622 | RPL22L1 |
| ENSG00000196634 | ENSG00000140743 | -0.7603 | CDR2 |
| ENSG00000255670 | ENSG00000271524 | -0.7602 | BNIP3P17 |
| ENSG00000255670 | ENSG00000274070 | -0.7544 | CASTOR2 |
| ENSG00000278905 | ENSG00000253779 | -0.7536 | IGLVVI-25-1 |
| ENSG00000196634 | ENSG00000225133 | -0.7520 | MORF4L1P4 |
| ENSG00000196634 | ENSG00000260230 | -0.7456 | FRRS1L |
| ENSG00000196634 | ENSG00000126787 | -0.7429 | DLGAP5 |
| ENSG00000278905 | ENSG00000015520 | -0.7423 | NPC1L1 |
| ENSG00000240980 | ENSG00000114098 | -0.7410 | ARMC8 |
| ENSG00000240980 | ENSG00000287338 | -0.7383 | AL929091.1 |
| ENSG00000196634 | ENSG00000151576 | -0.7345 | QTRT2 |
| ENSG00000278905 | ENSG00000227949 | -0.7276 | CYCSP46 |
| ENSG00000196634 | ENSG00000220739 | -0.7261 | AL513475.2 |
| ENSG00000278905 | ENSG00000100505 | -0.7230 | TRIM9 |
| ENSG00000240980 | ENSG00000272305 | -0.7228 | AC096887.1 |
| ENSG00000196634 | ENSG00000155096 | -0.7188 | AZIN1 |
| ENSG00000278905 | ENSG00000270367 | -0.7163 | AC092053.1 |
| ENSG00000196634 | ENSG00000001629 | -0.7162 | ANKIB1 |
| ENSG00000240980 | ENSG00000270424 | -0.7152 | AC145285.5 |
| ENSG00000278905 | ENSG00000172215 | -0.7147 | CXCR6 |
| ENSG00000278905 | ENSG00000134058 | -0.7146 | CDK7 |
| ENSG00000255670 | ENSG00000270890 | -0.7146 | AL049844.2 |
| ENSG00000255670 | ENSG00000025039 | -0.7112 | RRAGD |
| ENSG00000196634 | ENSG00000254634 | -0.7077 | SMG1P6 |
| ENSG00000278905 | ENSG00000235162 | -0.7066 | C12orf75 |
| ENSG00000196634 | ENSG00000166825 | -0.7061 | ANPEP |
| ENSG00000278905 | ENSG00000256937 | -0.7044 | KRT17P8 |
| ENSG00000278905 | ENSG00000152254 | -0.7041 | G6PC2 |
| ENSG00000255670 | ENSG00000120725 | -0.7028 | SIL1 |
| ENSG00000240980 | ENSG00000251019 | -0.7013 | HIGD1AP13 |
| ENSG00000240980 | ENSG00000189377 | -0.6982 | CXCL17 |
| ENSG00000196634 | ENSG00000233487 | -0.6967 | RPSAP69 |
| ENSG00000278905 | ENSG00000257757 | -0.6956 | OR6C7P |
| ENSG00000196634 | ENSG00000130244 | -0.6945 | FAM98C |
| ENSG00000278905 | ENSG00000225813 | -0.6937 | AC009299.1 |
| ENSG00000255670 | ENSG00000188818 | -0.6924 | ZDHHC11 |
| ENSG00000278905 | ENSG00000277406 | -0.6910 | SEC22B4P |
| ENSG00000255670 | ENSG00000257127 | -0.6899 | CLLU1 |
| ENSG00000240980 | ENSG00000287386 | -0.6889 | AC009248.3 |
| ENSG00000278905 | ENSG00000230951 | -0.6851 | GPS2P2 |
| ENSG00000278905 | ENSG00000284882 | -0.6836 | AL359762.1 |
| ENSG00000278905 | ENSG00000256223 | -0.6832 | ZNF10 |
| ENSG00000196634 | ENSG00000253032 | -0.6773 | RNU6-299P |
| ENSG00000278905 | ENSG00000288050 | -0.6769 | AC010727.1 |
| ENSG00000278905 | ENSG00000226491 | -0.6718 | FTOP1 |
| ENSG00000278905 | ENSG00000188227 | -0.6709 | ZNF793 |
| ENSG00000240980 | ENSG00000255815 | -0.6701 | KRT8P11 |
| ENSG00000196634 | ENSG00000266497 | -0.6699 | RDM1P2 |
| ENSG00000196634 | ENSG00000198851 | -0.6661 | CD3E |
| ENSG00000196634 | ENSG00000286092 | -0.6660 | AC117503.5 |
| ENSG00000240980 | ENSG00000188582 | -0.6628 | PAQR9 |
| ENSG00000255670 | ENSG00000286389 | -0.6582 | AL731702.1 |
| ENSG00000240980 | ENSG00000095587 | -0.6574 | TLL2 |
| ENSG00000196634 | ENSG00000259043 | -0.6556 | BRD7P1 |
| ENSG00000196634 | ENSG00000286970 | -0.6548 | AC010491.2 |
| ENSG00000240980 | ENSG00000243883 | -0.6542 | RN7SL419P |
| ENSG00000278905 | ENSG00000184232 | -0.6538 | OAF |
| ENSG00000278905 | ENSG00000109686 | -0.6529 | SH3D19 |
| ENSG00000278905 | ENSG00000188322 | -0.6516 | SBK1 |
| ENSG00000255670 | ENSG00000287330 | -0.6494 | AL353697.1 |
| ENSG00000278905 | ENSG00000177025 | -0.6406 | C19orf18 |
| ENSG00000255670 | ENSG00000248971 | -0.6397 | KRT8P46 |
| ENSG00000278905 | ENSG00000172954 | -0.6385 | LCLAT1 |
| ENSG00000196634 | ENSG00000276710 | -0.6357 | CSPG4P10 |
| ENSG00000255670 | ENSG00000254326 | -0.6356 | IGHV7-27 |
| ENSG00000278905 | ENSG00000267253 | -0.6316 | WHSC1L2P |
| ENSG00000240980 | ENSG00000257720 | -0.6280 | ILF2P2 |
| ENSG00000196634 | ENSG00000285287 | -0.6274 | AL354855.1 |
| ENSG00000278905 | ENSG00000238374 | -0.6260 | RNU7-180P |
| ENSG00000278905 | ENSG00000134152 | -0.6253 | KATNBL1 |
| ENSG00000240980 | ENSG00000269385 | -0.6249 | AC020895.1 |
| ENSG00000278905 | ENSG00000121486 | -0.6228 | TRMT1L |
| ENSG00000255670 | ENSG00000120694 | -0.6224 | HSPH1 |
| ENSG00000255670 | ENSG00000154079 | -0.6208 | SDHAF4 |
| ENSG00000196634 | ENSG00000135297 | -0.6159 | MTO1 |
| ENSG00000196634 | ENSG00000285728 | -0.6150 | AC098484.4 |
| ENSG00000278905 | ENSG00000125611 | -0.6135 | CHCHD5 |
| ENSG00000196634 | ENSG00000196597 | -0.6103 | ZNF782 |
| ENSG00000278905 | ENSG00000065923 | -0.6086 | SLC9A7 |
| ENSG00000278905 | ENSG00000234607 | -0.6069 | AL355994.4 |
| ENSG00000196634 | ENSG00000229462 | -0.6029 | AC127383.1 |
| ENSG00000196634 | ENSG00000124813 | -0.6028 | RUNX2 |
| ENSG00000255670 | ENSG00000267508 | -0.6010 | ZNF285 |
| ENSG00000196634 | ENSG00000182749 | -0.5995 | PAQR7 |
| ENSG00000240980 | ENSG00000104371 | -0.5988 | DKK4 |
| ENSG00000278905 | ENSG00000060971 | -0.5925 | ACAA1 |
| ENSG00000196634 | ENSG00000287978 | -0.5898 | AC245407.2 |
| ENSG00000196634 | ENSG00000286016 | -0.5878 | AC079949.3 |
| ENSG00000240980 | ENSG00000250815 | -0.5866 | AC105384.2 |
| ENSG00000278905 | ENSG00000139160 | -0.5865 | ETFBKMT |
| ENSG00000240980 | ENSG00000111696 | -0.5844 | NT5DC3 |
| ENSG00000196634 | ENSG00000079785 | -0.5823 | DDX1 |
| ENSG00000278905 | ENSG00000183688 | -0.5770 | RFLNB |
| ENSG00000255670 | ENSG00000145687 | -0.5767 | SSBP2 |
| ENSG00000255670 | ENSG00000255837 | -0.5755 | TAS2R20 |
| ENSG00000240980 | ENSG00000090339 | -0.5735 | ICAM1 |
| ENSG00000240980 | ENSG00000170500 | -0.5734 | LONRF2 |
| ENSG00000240980 | ENSG00000158716 | -0.5726 | DUSP23 |
| ENSG00000196634 | ENSG00000222268 | -0.5711 | RNA5SP425 |
| ENSG00000278905 | ENSG00000108469 | -0.5694 | RECQL5 |
| ENSG00000196634 | ENSG00000244264 | -0.5613 | RN7SL597P |
| ENSG00000278905 | ENSG00000254612 | -0.5580 | DNAJB6P1 |
| ENSG00000278905 | ENSG00000221946 | -0.5558 | FXYD7 |
| ENSG00000240980 | ENSG00000188997 | -0.5547 | KCTD21 |
| ENSG00000278905 | ENSG00000139351 | -0.5534 | SYCP3 |
| ENSG00000255670 | ENSG00000261594 | -0.5530 | TPBGL |
| ENSG00000278905 | ENSG00000212440 | -0.5512 | SNORA75 |
| ENSG00000278905 | ENSG00000287803 | -0.5504 | AC117569.2 |
| ENSG00000255670 | ENSG00000260870 | -0.5490 | NDUFB10P1 |
| ENSG00000278905 | ENSG00000196437 | -0.5471 | ZNF569 |
| ENSG00000196634 | ENSG00000196335 | -0.5417 | STK31 |
| ENSG00000196634 | ENSG00000235429 | -0.5398 | AC083875.1 |
| ENSG00000278905 | ENSG00000230104 | -0.5390 | AC018712.1 |
| ENSG00000278905 | ENSG00000172717 | -0.5350 | FAM71D |
| ENSG00000278905 | ENSG00000268058 | -0.5349 | BNIP3P40 |
| ENSG00000278905 | ENSG00000243101 | -0.5348 | RPS3P7 |
| ENSG00000278905 | ENSG00000173141 | -0.5340 | MRPL57 |
| ENSG00000278905 | ENSG00000212807 | -0.5335 | OR2A42 |
| ENSG00000278905 | ENSG00000137473 | -0.5324 | TTC29 |
| ENSG00000196634 | ENSG00000240024 | -0.5306 | LINC00888 |
| ENSG00000255670 | ENSG00000160345 | -0.5305 | C9orf116 |
| ENSG00000278905 | ENSG00000233264 | -0.5297 | AC006042.2 |
| ENSG00000278905 | ENSG00000161328 | -0.5281 | LRRC56 |
| ENSG00000278905 | ENSG00000130711 | -0.5280 | PRDM12 |
| ENSG00000196634 | ENSG00000287149 | -0.5271 | AC092640.1 |
| ENSG00000196634 | ENSG00000260865 | -0.5256 | AC010287.1 |
| ENSG00000255670 | ENSG00000144821 | -0.5252 | MYH15 |
| ENSG00000240980 | ENSG00000207296 | -0.5171 | RNU6-140P |
| ENSG00000255670 | ENSG00000137073 | -0.5165 | UBAP2 |
| ENSG00000278905 | ENSG00000204745 | -0.5162 | AC083899.1 |
| ENSG00000278905 | ENSG00000275451 | -0.5135 | MIR6085 |
| ENSG00000196634 | ENSG00000261739 | -0.5132 | GOLGA8S |
| ENSG00000278905 | ENSG00000231017 | -0.5131 | RPS27P10 |
| ENSG00000278905 | ENSG00000256817 | -0.5127 | TPT1P12 |
| ENSG00000196634 | ENSG00000181513 | -0.5100 | ACBD4 |
| ENSG00000196634 | ENSG00000183199 | -0.5076 | HSP90AB3P |
| ENSG00000255670 | ENSG00000212321 | -0.5045 | U3 |
| ENSG00000278905 | ENSG00000153006 | -0.5044 | SREK1IP1 |
| ENSG00000278905 | ENSG00000171094 | -0.5038 | ALK |
| ENSG00000196634 | ENSG00000286466 | -0.5034 | AC069280.2 |
| ENSG00000255670 | ENSG00000139266 | -0.5004 | MARCHF9 |
| ENSG00000240980 | ENSG00000039650 | 0.5011 | PNKP |
| ENSG00000278905 | ENSG00000257687 | 0.5019 | AC079600.2 |
| ENSG00000196634 | ENSG00000220960 | 0.5020 | AL079342.2 |
| ENSG00000278905 | ENSG00000225932 | 0.5021 | CTAGE4 |
| ENSG00000196634 | ENSG00000132801 | 0.5033 | ZSWIM3 |
| ENSG00000240980 | ENSG00000236187 | 0.5035 | GJA6P |
| ENSG00000196634 | ENSG00000069696 | 0.5044 | DRD4 |
| ENSG00000255670 | ENSG00000198054 | 0.5048 | DSCR8 |
| ENSG00000278905 | ENSG00000025770 | 0.5054 | NCAPH2 |
| ENSG00000240980 | ENSG00000130656 | 0.5075 | HBZ |
| ENSG00000240980 | ENSG00000150457 | 0.5078 | LATS2 |
| ENSG00000240980 | ENSG00000104953 | 0.5091 | TLE6 |
| ENSG00000240980 | ENSG00000142556 | 0.5099 | ZNF614 |
| ENSG00000196634 | ENSG00000244378 | 0.5102 | RPS2P45 |
| ENSG00000278905 | ENSG00000202058 | 0.5108 | RN7SKP80 |
| ENSG00000278905 | ENSG00000228259 | 0.5114 | AC136604.1 |
| ENSG00000255670 | ENSG00000143067 | 0.5124 | ZNF697 |
| ENSG00000240980 | ENSG00000230832 | 0.5128 | AC241584.1 |
| ENSG00000255670 | ENSG00000213421 | 0.5131 | AC099670.1 |
| ENSG00000240980 | ENSG00000166340 | 0.5158 | TPP1 |
| ENSG00000240980 | ENSG00000204887 | 0.5174 | KRTAP1-4 |
| ENSG00000240980 | ENSG00000233549 | 0.5174 | CYCSP35 |
| ENSG00000278905 | ENSG00000184492 | 0.5175 | FOXD4L1 |
| ENSG00000255670 | ENSG00000164574 | 0.5179 | GALNT10 |
| ENSG00000255670 | ENSG00000134864 | 0.5181 | GGACT |
| ENSG00000255670 | ENSG00000111249 | 0.5188 | CUX2 |
| ENSG00000278905 | ENSG00000150773 | 0.5192 | PIH1D2 |
| ENSG00000196634 | ENSG00000120314 | 0.5195 | WDR55 |
| ENSG00000196634 | ENSG00000100058 | 0.5196 | CRYBB2P1 |
| ENSG00000255670 | ENSG00000198920 | 0.5211 | KIAA0753 |
| ENSG00000240980 | ENSG00000253954 | 0.5217 | HMGN1P38 |
| ENSG00000196634 | ENSG00000224312 | 0.5242 | MCCD1P2 |
| ENSG00000255670 | ENSG00000166787 | 0.5278 | SAA3P |
| ENSG00000196634 | ENSG00000260318 | 0.5297 | COX6CP1 |
| ENSG00000255670 | ENSG00000184831 | 0.5318 | APOO |
| ENSG00000278905 | ENSG00000146376 | 0.5328 | ARHGAP18 |
| ENSG00000278905 | ENSG00000211789 | 0.5328 | TRAV12-2 |
| ENSG00000240980 | ENSG00000152952 | 0.5334 | PLOD2 |
| ENSG00000196634 | ENSG00000106483 | 0.5350 | SFRP4 |
| ENSG00000240980 | ENSG00000164303 | 0.5384 | ENPP6 |
| ENSG00000196634 | ENSG00000196090 | 0.5396 | PTPRT |
| ENSG00000255670 | ENSG00000206907 | 0.5403 | RNU6-1013P |
| ENSG00000278905 | ENSG00000267952 | 0.5407 | AC008878.1 |
| ENSG00000196634 | ENSG00000227454 | 0.5436 | MTND4P30 |
| ENSG00000255670 | ENSG00000179240 | 0.5440 | GVQW3 |
| ENSG00000196634 | ENSG00000199788 | 0.5441 | RNY3P2 |
| ENSG00000255670 | ENSG00000286044 | 0.5446 | AP001977.1 |
| ENSG00000255670 | ENSG00000202137 | 0.5447 | Y_RNA |
| ENSG00000255670 | ENSG00000130529 | 0.5454 | TRPM4 |
| ENSG00000255670 | ENSG00000184995 | 0.5484 | IFNE |
| ENSG00000240980 | ENSG00000151090 | 0.5511 | THRB |
| ENSG00000255670 | ENSG00000076662 | 0.5523 | ICAM3 |
| ENSG00000255670 | ENSG00000234109 | 0.5536 | RPL7P36 |
| ENSG00000196634 | ENSG00000147041 | 0.5539 | SYTL5 |
| ENSG00000255670 | ENSG00000199646 | 0.5541 | RNU6-1272P |
| ENSG00000255670 | ENSG00000162068 | 0.5563 | NTN3 |
| ENSG00000196634 | ENSG00000265078 | 0.5570 | RN7SL664P |
| ENSG00000255670 | ENSG00000207756 | 0.5573 | MIR580 |
| ENSG00000196634 | ENSG00000233609 | 0.5580 | RPL10P19 |
| ENSG00000255670 | ENSG00000264311 | 0.5598 | CCDC58P1 |
| ENSG00000240980 | ENSG00000171681 | 0.5602 | ATF7IP |
| ENSG00000255670 | ENSG00000196131 | 0.5604 | VN1R2 |
| ENSG00000196634 | ENSG00000240036 | 0.5609 | AC104563.1 |
| ENSG00000255670 | ENSG00000275607 | 0.5609 | AC135507.2 |
| ENSG00000240980 | ENSG00000216639 | 0.5670 | AL133406.1 |
| ENSG00000196634 | ENSG00000200818 | 0.5676 | RNU6-1204P |
| ENSG00000278905 | ENSG00000166090 | 0.5677 | IL25 |
| ENSG00000196634 | ENSG00000204869 | 0.5697 | IGFL4 |
| ENSG00000278905 | ENSG00000099282 | 0.5703 | TSPAN15 |
| ENSG00000240980 | ENSG00000130177 | 0.5732 | CDC16 |
| ENSG00000196634 | ENSG00000228367 | 0.5733 | AC108120.1 |
| ENSG00000196634 | ENSG00000244361 | 0.5740 | RPL30P7 |
| ENSG00000196634 | ENSG00000106565 | 0.5742 | TMEM176B |
| ENSG00000196634 | ENSG00000143375 | 0.5794 | CGN |
| ENSG00000255670 | ENSG00000173221 | 0.5810 | GLRX |
| ENSG00000240980 | ENSG00000145901 | 0.5812 | TNIP1 |
| ENSG00000278905 | ENSG00000237827 | 0.5812 | RPS15AP29 |
| ENSG00000255670 | ENSG00000219294 | 0.5823 | PIP5K1P1 |
| ENSG00000255670 | ENSG00000277149 | 0.5825 | TYW1B |
| ENSG00000240980 | ENSG00000010244 | 0.5834 | ZNF207 |
| ENSG00000196634 | ENSG00000170315 | 0.5834 | UBB |
| ENSG00000196634 | ENSG00000231390 | 0.5853 | SNX18P8 |
| ENSG00000278905 | ENSG00000134056 | 0.5863 | MRPS36 |
| ENSG00000278905 | ENSG00000265648 | 0.5867 | RN7SL279P |
| ENSG00000196634 | ENSG00000159761 | 0.5869 | C16orf86 |
| ENSG00000240980 | ENSG00000128463 | 0.5920 | EMC4 |
| ENSG00000240980 | ENSG00000088727 | 0.5926 | KIF9 |
| ENSG00000196634 | ENSG00000131015 | 0.5931 | ULBP2 |
| ENSG00000278905 | ENSG00000243896 | 0.5936 | OR2A7 |
| ENSG00000196634 | ENSG00000150048 | 0.5971 | CLEC1A |
| ENSG00000278905 | ENSG00000111261 | 0.5976 | MANSC1 |
| ENSG00000240980 | ENSG00000173674 | 0.5981 | EIF1AX |
| ENSG00000196634 | ENSG00000249372 | 0.6016 | ATP6V1G1P6 |
| ENSG00000240980 | ENSG00000287495 | 0.6018 | AL356489.4 |
| ENSG00000240980 | ENSG00000250305 | 0.6084 | TRMT9B |
| ENSG00000255670 | ENSG00000111144 | 0.6094 | LTA4H |
| ENSG00000278905 | ENSG00000166068 | 0.6113 | SPRED1 |
| ENSG00000240980 | ENSG00000114626 | 0.6166 | ABTB1 |
| ENSG00000196634 | ENSG00000264722 | 0.6181 | MIR3670-2 |
| ENSG00000240980 | ENSG00000164638 | 0.6183 | SLC29A4 |
| ENSG00000255670 | ENSG00000006638 | 0.6207 | TBXA2R |
| ENSG00000278905 | ENSG00000144395 | 0.6213 | CCDC150 |
| ENSG00000196634 | ENSG00000013725 | 0.6241 | CD6 |
| ENSG00000255670 | ENSG00000085365 | 0.6257 | SCAMP1 |
| ENSG00000278905 | ENSG00000115138 | 0.6264 | POMC |
| ENSG00000196634 | ENSG00000271410 | 0.6285 | AC020728.1 |
| ENSG00000278905 | ENSG00000077514 | 0.6302 | POLD3 |
| ENSG00000255670 | ENSG00000201379 | 0.6354 | RNU4-76P |
| ENSG00000255670 | ENSG00000127423 | 0.6373 | AUNIP |
| ENSG00000255670 | ENSG00000132746 | 0.6376 | ALDH3B2 |
| ENSG00000278905 | ENSG00000131059 | 0.6377 | BPIFA3 |
| ENSG00000278905 | ENSG00000168438 | 0.6409 | CDC40 |
| ENSG00000255670 | ENSG00000165973 | 0.6410 | NELL1 |
| ENSG00000255670 | ENSG00000167705 | 0.6419 | RILP |
| ENSG00000278905 | ENSG00000276717 | 0.6441 | PRSS47 |
| ENSG00000240980 | ENSG00000127780 | 0.6470 | OR1E2 |
| ENSG00000278905 | ENSG00000102172 | 0.6527 | SMS |
| ENSG00000196634 | ENSG00000131652 | 0.6546 | THOC6 |
| ENSG00000255670 | ENSG00000161265 | 0.6551 | U2AF1L4 |
| ENSG00000196634 | ENSG00000118200 | 0.6553 | CAMSAP2 |
| ENSG00000255670 | ENSG00000133612 | 0.6562 | AGAP3 |
| ENSG00000278905 | ENSG00000252429 | 0.6564 | RNU7-29P |
| ENSG00000255670 | ENSG00000113532 | 0.6564 | ST8SIA4 |
| ENSG00000255670 | ENSG00000117640 | 0.6593 | MTFR1L |
| ENSG00000196634 | ENSG00000271269 | 0.6611 | AL353778.1 |
| ENSG00000196634 | ENSG00000134709 | 0.6614 | HOOK1 |
| ENSG00000255670 | ENSG00000205822 | 0.6635 | TPTE2P6 |
| ENSG00000196634 | ENSG00000173200 | 0.6674 | PARP15 |
| ENSG00000278905 | ENSG00000174130 | 0.6691 | TLR6 |
| ENSG00000255670 | ENSG00000232174 | 0.6709 | AC113340.1 |
| ENSG00000196634 | ENSG00000233623 | 0.6713 | PGAM1P11 |
| ENSG00000255670 | ENSG00000207619 | 0.6725 | MIR585 |
| ENSG00000240980 | ENSG00000252200 | 0.6751 | AC243964.2 |
| ENSG00000240980 | ENSG00000232716 | 0.6754 | AC016831.3 |
| ENSG00000240980 | ENSG00000184619 | 0.6841 | KRBA2 |
| ENSG00000278905 | ENSG00000158552 | 0.6843 | ZFAND2B |
| ENSG00000255670 | ENSG00000169093 | 0.6928 | ASMTL |
| ENSG00000196634 | ENSG00000115661 | 0.6984 | STK16 |
| ENSG00000196634 | ENSG00000087086 | 0.6989 | FTL |
| ENSG00000278905 | ENSG00000147669 | 0.6991 | POLR2K |
| ENSG00000255670 | ENSG00000106211 | 0.7010 | HSPB1 |
| ENSG00000255670 | ENSG00000166780 | 0.7028 | BMERB1 |
| ENSG00000240980 | ENSG00000109101 | 0.7047 | FOXN1 |
| ENSG00000196634 | ENSG00000287180 | 0.7068 | AC106818.2 |
| ENSG00000196634 | ENSG00000181323 | 0.7076 | SPEM1 |
| ENSG00000196634 | ENSG00000229150 | 0.7089 | CRYGEP |
| ENSG00000278905 | ENSG00000250507 | 0.7150 | AC245884.6 |
| ENSG00000278905 | ENSG00000105708 | 0.7185 | ZNF14 |
| ENSG00000196634 | ENSG00000159885 | 0.7244 | ZNF222 |
| ENSG00000278905 | ENSG00000174939 | 0.7259 | ASPHD1 |
| ENSG00000196634 | ENSG00000204711 | 0.7265 | C9orf135 |
| ENSG00000255670 | ENSG00000066739 | 0.7282 | ATG2B |
| ENSG00000255670 | ENSG00000142192 | 0.7283 | APP |
| ENSG00000278905 | ENSG00000121741 | 0.7305 | ZMYM2 |
| ENSG00000278905 | ENSG00000286020 | 0.7336 | AC011997.2 |
| ENSG00000255670 | ENSG00000108306 | 0.7446 | FBXL20 |
| ENSG00000196634 | ENSG00000198863 | 0.7478 | RUNDC1 |
| ENSG00000196634 | ENSG00000134815 | 0.7514 | DHX34 |
| ENSG00000196634 | ENSG00000140320 | 0.7538 | BAHD1 |
| ENSG00000278905 | ENSG00000184140 | 0.7588 | OR4F6 |
| ENSG00000196634 | ENSG00000206106 | 0.7618 | KRTAP22-2 |
| ENSG00000278905 | ENSG00000100592 | 0.7700 | DAAM1 |
| ENSG00000240980 | ENSG00000182400 | 0.7716 | TRAPPC6B |
| ENSG00000196634 | ENSG00000241926 | 0.7752 | MTCO1P55 |
| ENSG00000196634 | ENSG00000273024 | 0.7752 | INTS4P2 |
| ENSG00000196634 | ENSG00000188566 | 0.7776 | NDOR1 |
| ENSG00000278905 | ENSG00000176009 | 0.7789 | ASCL3 |
| ENSG00000196634 | ENSG00000227609 | 0.7817 | TMEM183AP1 |
| ENSG00000196634 | ENSG00000177590 | 0.7823 | GIMAP3P |
| ENSG00000278905 | ENSG00000276650 | 0.7897 | AC022202.1 |
| ENSG00000196634 | ENSG00000106028 | 0.7897 | SSBP1 |
| ENSG00000278905 | ENSG00000105697 | 0.7925 | HAMP |
| ENSG00000278905 | ENSG00000105835 | 0.7936 | NAMPT |
| ENSG00000196634 | ENSG00000167637 | 0.8002 | ZNF283 |
| ENSG00000196634 | ENSG00000102010 | 0.8058 | BMX |
| ENSG00000196634 | ENSG00000227999 | 0.8129 | MTND5P1 |
| ENSG00000240980 | ENSG00000210107 | 0.8147 | MT-TQ |
| ENSG00000278905 | ENSG00000107438 | 0.8149 | PDLIM1 |
| ENSG00000196634 | ENSG00000165076 | 0.8197 | PRSS37 |
| ENSG00000196634 | ENSG00000164402 | 0.8198 | SEPTIN8 |
| ENSG00000196634 | ENSG00000138785 | 0.8246 | INTS12 |
| ENSG00000278905 | ENSG00000152229 | 0.8326 | PSTPIP2 |
| ENSG00000196634 | ENSG00000132953 | 0.8338 | XPO4 |
| ENSG00000278905 | ENSG00000237172 | 0.8447 | B3GNT9 |
| ENSG00000196634 | ENSG00000197557 | 0.8584 | TTC30A |
| ENSG00000278905 | ENSG00000105251 | 0.8647 | SHD |
| ENSG00000278905 | ENSG00000287337 | 0.8686 | AC005962.2 |
| ENSG00000196634 | ENSG00000111341 | 0.8710 | MGP |
| ENSG00000278905 | ENSG00000074964 | 0.8733 | ARHGEF10L |
| ENSG00000278905 | ENSG00000157214 | 0.8788 | STEAP2 |
| ENSG00000278905 | ENSG00000108179 | 0.8862 | PPIF |
| ENSG00000196634 | ENSG00000100296 | 0.8868 | THOC5 |
